# Supplementary material for: Single-cell analysis highlights differences in druggable pathways underlying adaptive or fibrotic kidney regeneration
Source: Nat Commun. 2022 Jul 11;13:4018. doi: 10.1038/s41467-022-31772-9 (PMC9276703; doi:10.1038/s41467-022-31772-9)
Supplement: Supplementary file 1 — Supplementary Information [file 41467_2022_31772_MOESM1_ESM.pdf]

Supplementary Information

Single-cell analysis highlights differences in druggable pathways underlying adaptive or maladaptive kidney regeneration

Michael S. Balzer[1,2], Tomohito Doke[1,2], Ya-Wen Yang[1,2], Daniel L. Aldridge[3], Hailong Hu[1,2], Hung Mai[1,2], Dhanunjay Mukhi[1,2], Ziyuan Ma[1,2], Rojesh Shrestha[1,2], Matthew B. Palmer[4], Christopher A. Hunter[3], and Katalin Susztak[1,2,5,*]

[1]Renal, Electrolyte, and Hypertension Division, Department of Medicine, Perelman School of Medicine, University of Pennsylvania, Philadelphia, PA 19104, USA
[2]Institute for Diabetes, Obesity and Metabolism, Perelman School of Medicine, University of Pennsylvania, Philadelphia, PA 19104, USA
[3]Department of Pathobiology, School of Veterinary Medicine, University of Pennsylvania, Philadelphia, PA 19104
[4]Department of Pathology and Laboratory Medicine, Perelman School of Medicine, University of Pennsylvania, Philadelphia, PA 19104
[5]Department of Genetics, Perelman School of Medicine, University of Pennsylvania, Philadelphia, PA 19104, USA

***Correspondence:**
Katalin Susztak, MD, PhD
ksusztak@pennmedicine.upenn.edu

Supplementary Figures

Pages 2-44

Supplementary Tables

Pages 45-47

Fig. S1

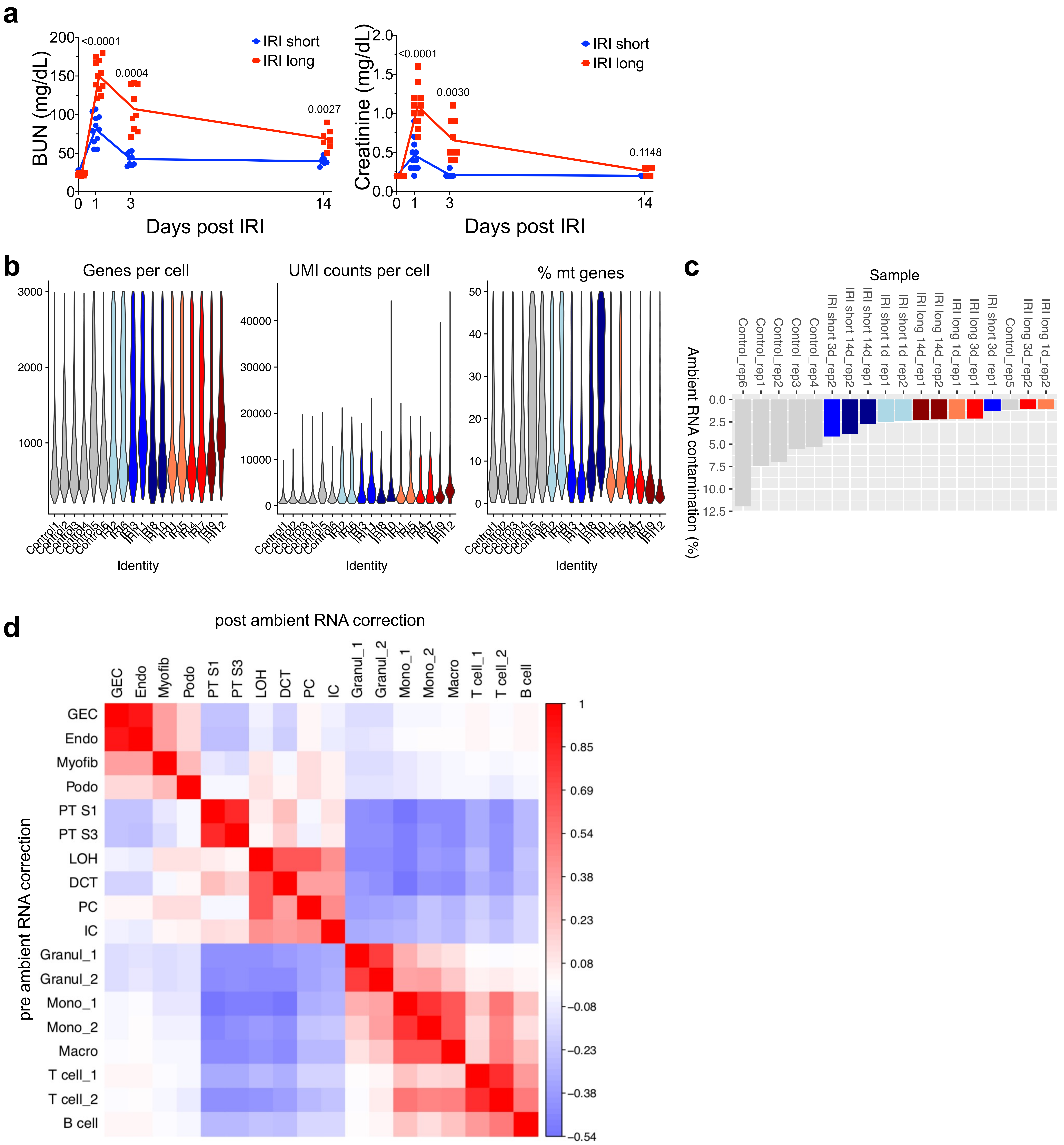
a
BUN (mg/dL)
IRI short
IRI long
<0.0001
0.0004
0.0027
Days post IRI
Creatinine (mg/dL)
<0.0001
0.0030
0.1148
Days post IRI
b
Genes per cell
UMI counts per cell
% mt genes
Identity
c
Sample
Control_rep6
Control_rep1
Control_rep2
Control_rep3
Control_rep4
IRI short 3d_rep2
IRI short 14d_rep2
IRI short 14d_rep1
IRI short 1d_rep1
IRI short 1d_rep2
IRI long 14d_rep1
IRI long 14d_rep2
IRI long 1d_rep1
IRI long 3d_rep1
IRI short 3d_rep1
Control_rep5
IRI long 3d_rep2
IRI long 1d_rep2
Ambient RNA contamination (%)
d
post ambient RNA correction
pre ambient RNA correction
GEC
Endo
Myofib
Podo
PT S1
PT S3
LOH
DCT
PC
IC
Granul_1
Granul_2
Mono_1
Mono_2
Macro
T cell_1
T cell_2
B cell

Figure S1. Ischemia reperfusion injury model titration & scRNA-seq quality control.

(a) BUN and blood creatinine 1, 3, and 14d post-IRI. The figure includes additional animals not used for scRNA-seq and bulk RNA-seq analysis, whereas **Fig. 1b** shows only animals used for scRNA-seq. p values are given for mixed-effects model (Sidak corrected) across n=5 independent experiments.

(b) Violin plots showing number of informative genes per single cell, unique molecular identifiers (UMIs) per single cell and percentage of mitochondrial genes per single cell, all split by batches. Dotted red lines indicate quality control limits and thresholds, respectively.

(c) Bar graph demonstrating the percentage of ambient RNA contamination per individual sample replicates, as estimated by SoupX. Gray bars denote Control samples, blue bars IRI short, red bars IRI long.

(d) Correlation matrix visualizing Pearson correlation coefficients (PCC) between average cell type gene expressions prior to (rows) and post (columns) correction for ambient RNA using SoupX.

Fig. S2

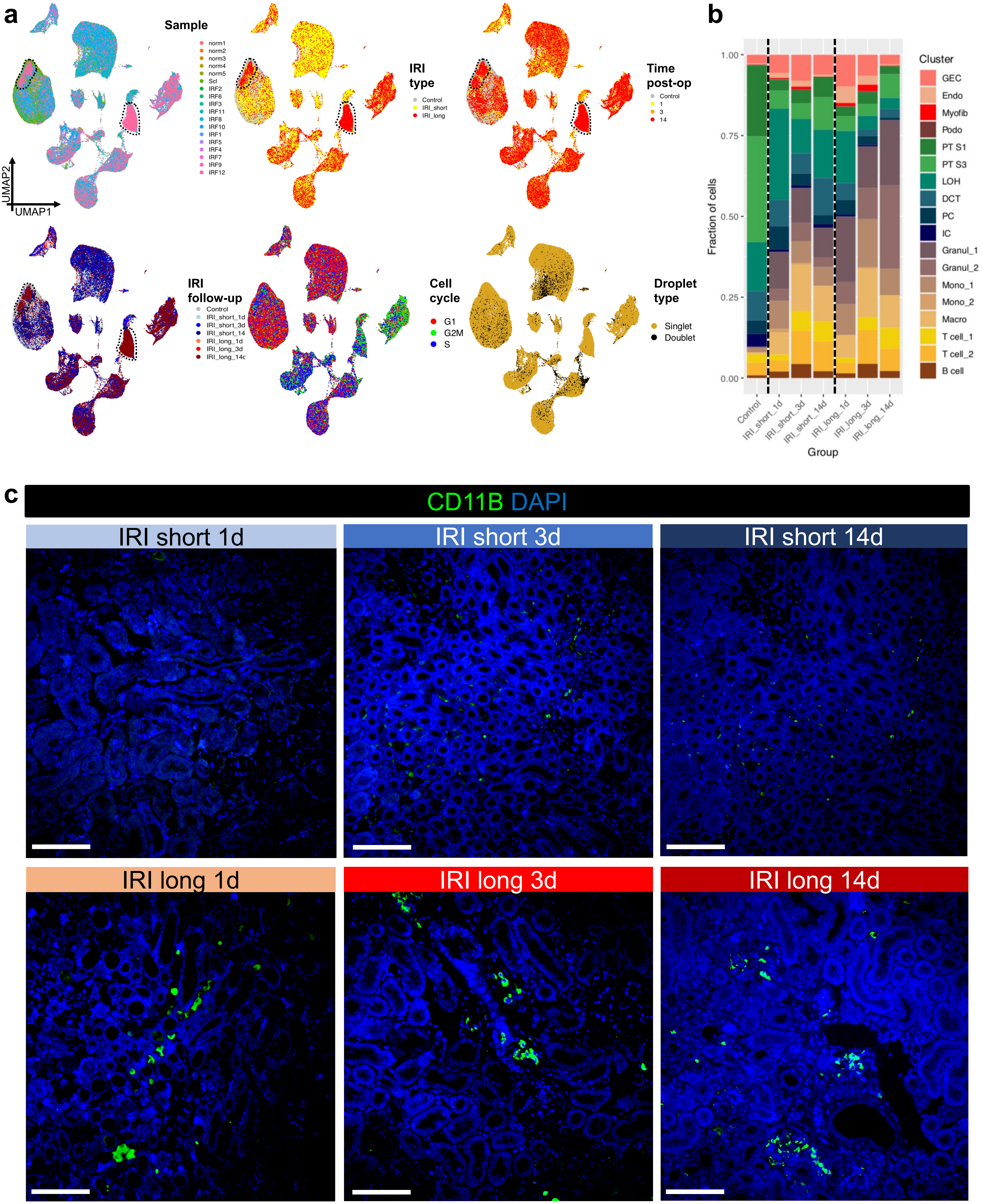
a
Sample
norm1
norm2
norm3
norm4
norm5
Scl
IRF2
IRF6
IRF3
IRF11
IRF8
IRF10
IRF1
IRF5
IRF4
IRF7
IRF9
IRF12
UMAP2
UMAP1
IRI type
Control
IRI_short
IRI_long
Time post-op
Control
1
3
14
IRI follow-up
Control
IRI_short_1d
IRI_short_3d
IRI_short_14
IRI_long_1d
IRI_long_3d
IRI_long_14c
Cell cycle
G1
G2M
S
Droplet type
Singlet
Doublet
b
Fraction of cells
1.00
0.75
0.50
0.25
0.00
Control
IRI_short_1d
IRI_short_3d
IRI_short_14d
IRI_long_1d
IRI_long_3d
IRI_long_14d
Group
Cluster
GEC
Endo
Myofib
Podo
PT S1
PT S3
LOH
DCT
PC
IC
Granul_1
Granul_2
Mono_1
Mono_2
Macro
T cell_1
T cell_2
B cell
c
CD11B DAPI
IRI short 1d
IRI short 3d
IRI short 14d
IRI long 1d
IRI long 3d
IRI long 14d

Figure S2. scRNA-seq cell type distribution, principal component heterogeneity, and myeloid cell infiltration.

(a) UMAP projection of 113,579 cells of n=6 controls, n=6 short IRI and n=6 long IRI samples colored by sample, IRI type (Control, short IRI, long IRI), time post-op (Control, 1d, 3d, 14d), IRI follow-up (Control, IRI short 1d, IRI short 3d, IRI short 14d, IRI long 1d, IRI long 3d, IRI long 14d), cell cycle phase (G1, G2M, S) as well as projection of 122,973 cells colored by droplet type (9,394 doublets were excluded in all other plots).

(b) Stacked bar graph showing the fraction of different cell types across different treatment groups and time points, respectively (Control, IRI short 1d, IRI short 3d, IRI short 14d, IRI long 1d, IRI long 3d, IRI long 14d): GEC, glomerular endothelial cell; Endo, endothelial cell; Myofib, myofibroblast; Podo, podocyte; PT S1, proximal convoluted tubule; PT S3, proximal straight tubule; LOH, loop of Henle; DCT, distal convoluted tubule; PC, principal cell; IC, intercalated cell; Granul, granulocyte; Mono, monocyte; Macro, macrophage; T cell; B cell.

(c) Immunofluorescence representative of n=3 independent experiments staining for myeloid cell marker CD11B; scale bars=50 μm.

Fig. S3

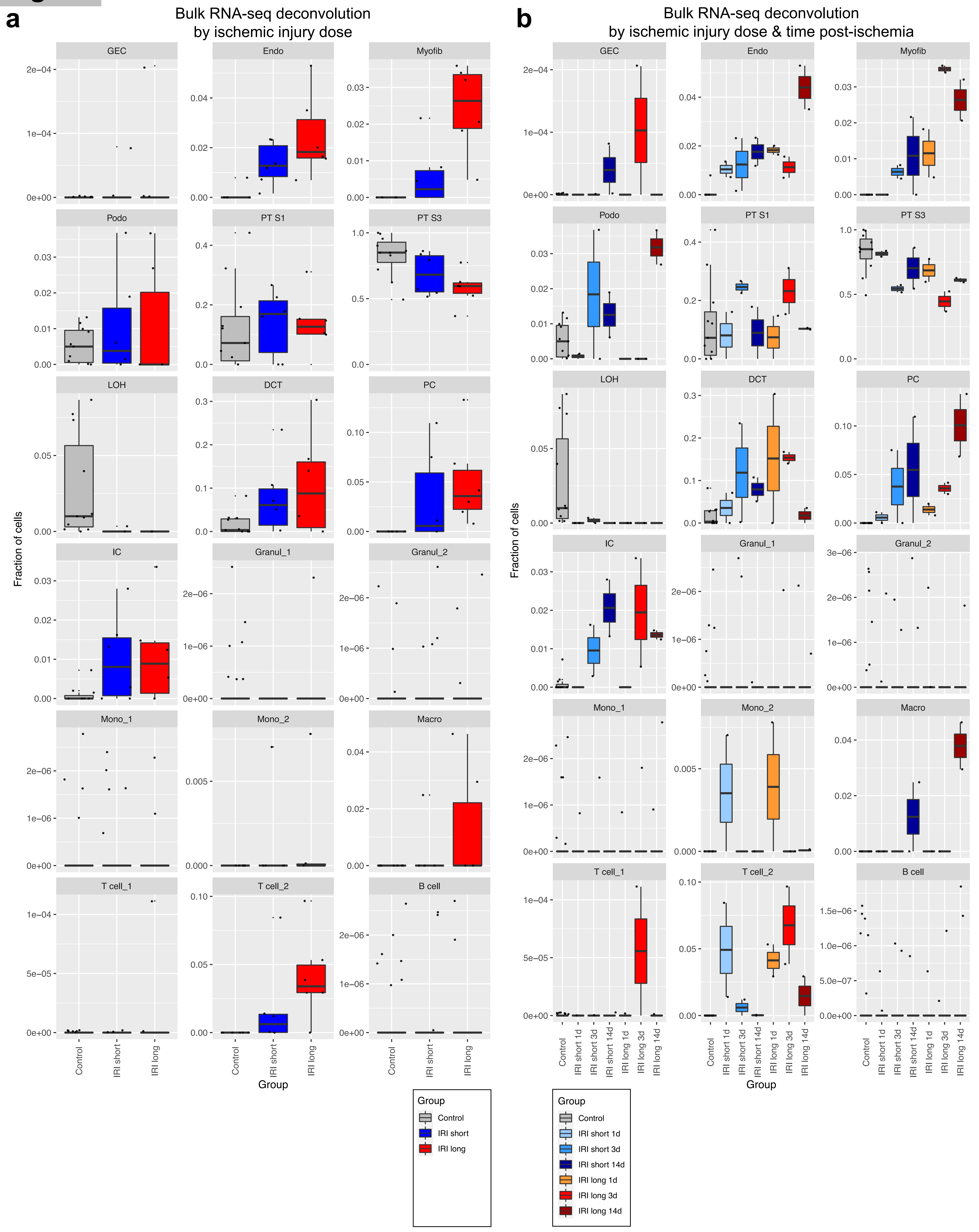
a
Bulk RNA-seq deconvolution by ischemic injury dose
GEC
Endo
Myofib
Podo
PT S1
PT S3
LOH
DCT
PC
IC
Granul_1
Granul_2
Mono_1
Mono_2
Macro
T cell_1
T cell_2
B cell
Fraction of cells
Group
Control
IRI short
IRI long
b
Bulk RNA-seq deconvolution by ischemic injury dose & time post-ischemia
Control
IRI short 1d
IRI short 3d
IRI short 14d
IRI long 1d
IRI long 3d
IRI long 14d

Figure S3. Bulk kidney RNA-seq deconvolution by IRI dose and time post-IRI.

(a-b) Cell fractions displayed as Tukey box plots after deconvolution of bulk RNA-seq data, stratified by injury dose only **(a)** and both injury dose and time post-ischemia ischemia **(b)**, respectively.

Fig. S4

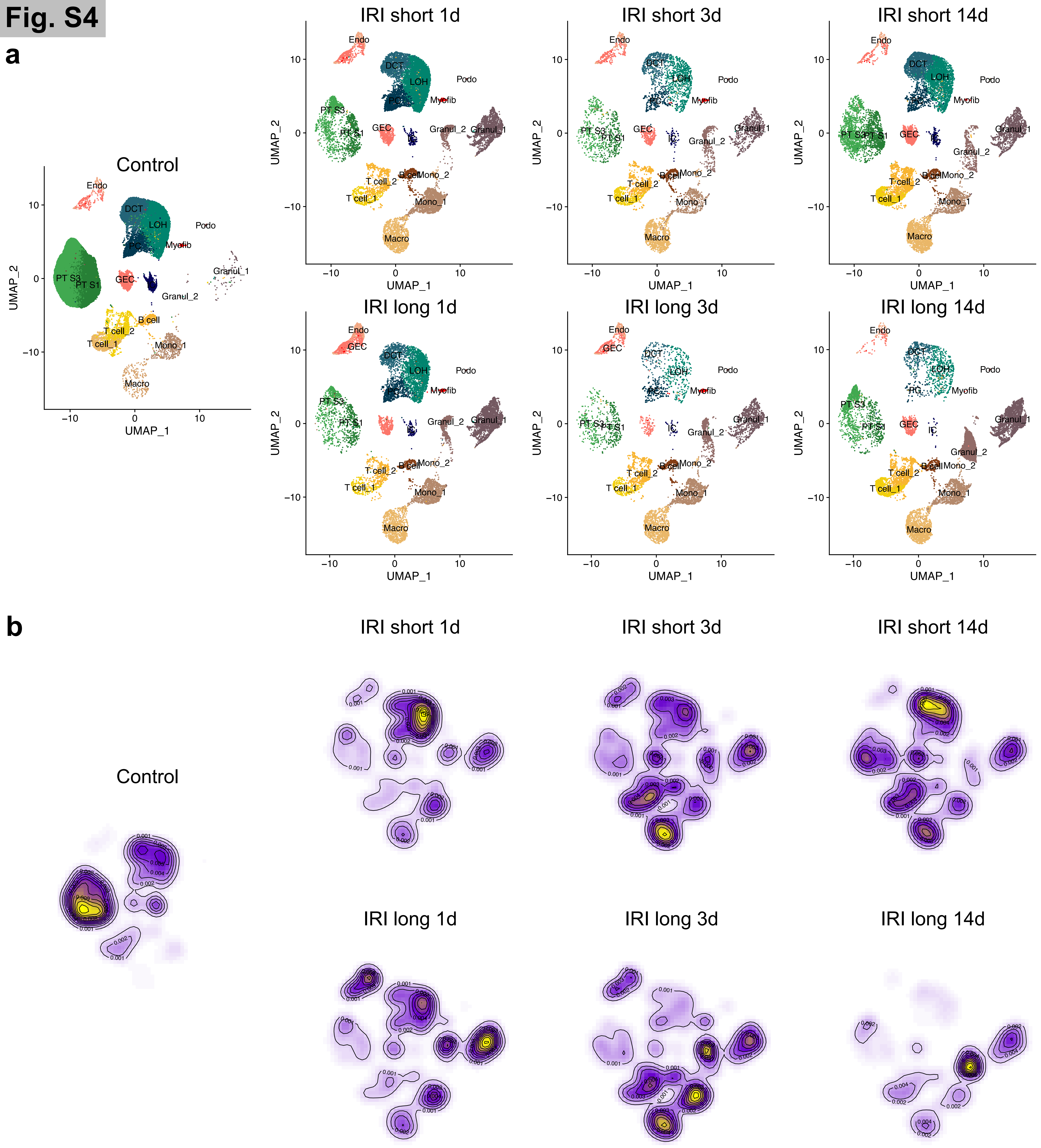
a
Control
IRI short 1d
IRI short 3d
IRI short 14d
IRI long 1d
IRI long 3d
IRI long 14d
UMAP_1
UMAP_2
Endo
DCT
LOH
Podo
PC
Myofib
PT S3
PT S1
GEC
IC
Granul_1
Granul_2
B cell
T cell_1
T cell_2
Mono_1
Mono_2
Macro
b
Control
IRI short 1d
IRI short 3d
IRI short 14d
IRI long 1d
IRI long 3d
IRI long 14d

Figure S4. Cell clusters and cell density stratified by injury dose and time post-ischemia.

(a-b) UMAP projections **(a)** and density plots **(b)** corresponding to **Figs. 1g** and **1k**, respectively, stratified by injury dose and time post-ischemia.

Fig. S5

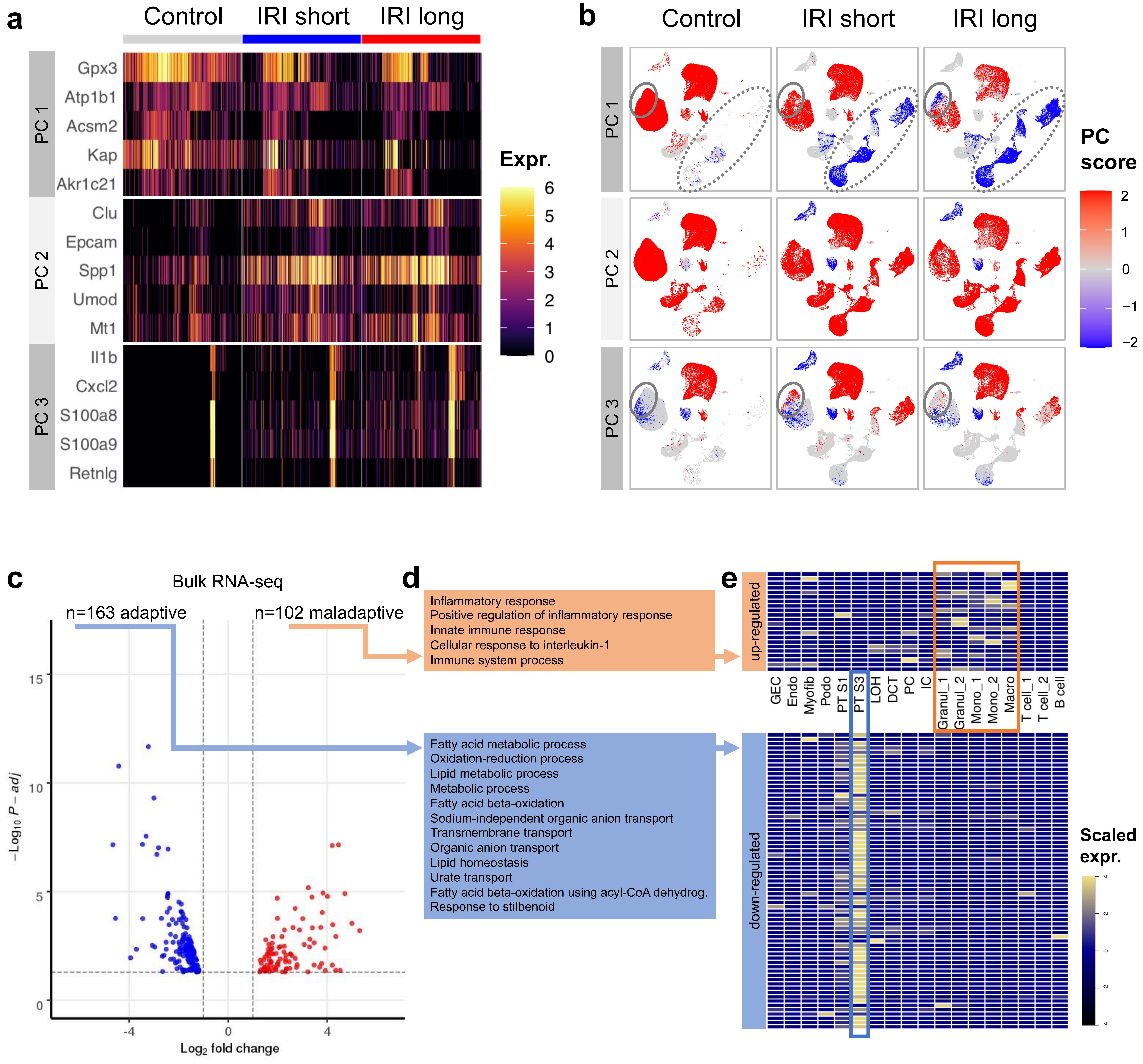
a
Control
IRI short
IRI long
PC 1
Gpx3
Atp1b1
Acsm2
Kap
Akr1c21
PC 2
Clu
Epcam
Spp1
Umod
Mt1
PC 3
Il1b
Cxcl2
S100a8
S100a9
Retnlg
Expr.
6
5
4
3
2
1
0
b
Control
IRI short
IRI long
PC 1
PC 2
PC 3
PC score
2
1
0
−1
−2
c
Bulk RNA-seq
n=163 adaptive
n=102 maladaptive
15
10
5
0
−4
0
4
Log2 fold change
−Log10 P − adj
d
Inflammatory response
Positive regulation of inflammatory response
Innate immune response
Cellular response to interleukin-1
Immune system process
Fatty acid metabolic process
Oxidation-reduction process
Lipid metabolic process
Metabolic process
Fatty acid beta-oxidation
Sodium-independent organic anion transport
Transmembrane transport
Organic anion transport
Lipid homeostasis
Urate transport
Fatty acid beta-oxidation using acyl-CoA dehydrog.
Response to stilbenoid
e
up-regulated
down-regulated
GEC
Endo
Myofib
Podo
PT S1
PT S3
LOH
DCT
PC
IC
Granul_1
Granul_2
Mono_1
Mono_2
Macro
T cell_1
T cell_2
B cell
Scaled expr.

Figure S5. scRNA-seq principal component heterogeneity, and bulk RNA-seq deconvolution.

(a) Heatmap visualizing the expression of top high-loading genes for the first three principal components (PC) based on PC analysis of the integrated dataset, illustrating recurrent aspects of transcriptional heterogeneity between treatment conditions (Control; IRI short; IRI long).

(b) Corresponding projections of scaled and normalized PC scores across different treatment conditions. The first 3 PCs are visualized. Maladapted PT cells (circle) and myeloid cells (dotted circle) contributed the most to PC heterogeneity.

(c) Volcano plot of differentially expressed genes (DEGs) comparing bulk RNA-seq data from samples 14d after short and long IRI, respectively. Genes upregulated in maladaptive samples are colored red, those in adaptive samples are colored blue. X axis indicates $\log_2$-fold change for two-tailed Wald test and y axis indicates statistical significance-adjusted p = $-\log_{10}$.

(d) Corresponding Gene Ontology (GO) analysis showing statistically significant GO terms corresponding to up- and downregulated DEGs from **(h)**.

(e) Heatmap showing scaled cell type-specific expression of top DEGs identified in bulk RNA-seq analysis from **(h)** in the scRNA-seq dataset. Mean expression values of the genes were calculated for each cluster.

Fig. S6

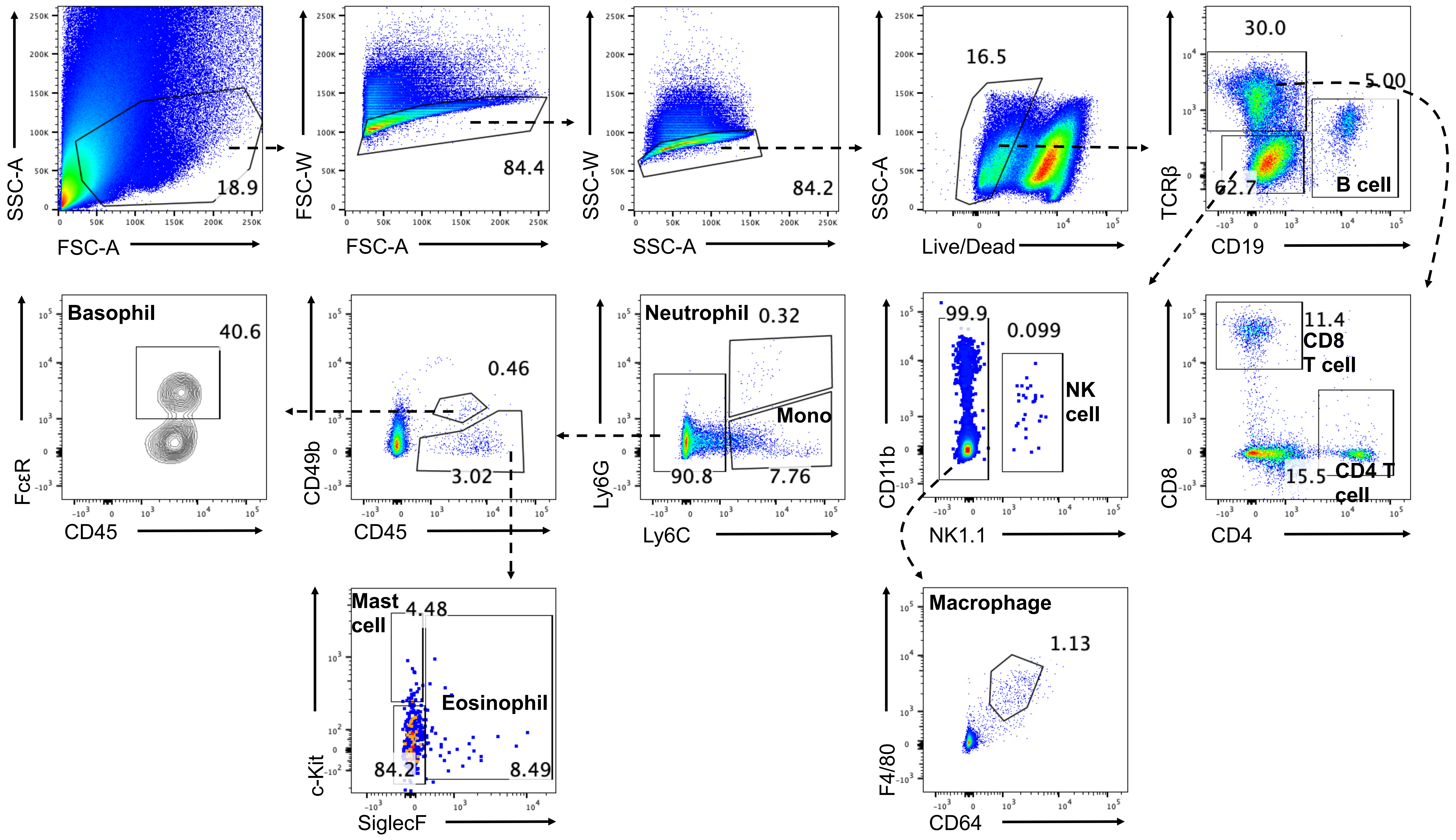

SSC-A
FSC-A
18.9
FSC-W
FSC-A
84.4
SSC-W
SSC-A
84.2
SSC-A
Live/Dead
16.5
TCRβ
CD19
30.0
5.00
62.7
B cell
FcεR
CD45
Basophil
40.6
CD49b
CD45
0.46
3.02
Ly6G
Ly6C
Neutrophil
0.32
Mono
90.8
7.76
CD11b
NK1.1
99.9
0.099
NK cell
CD8
CD4
11.4
CD8 T cell
15.5
CD4 T cell
c-Kit
SiglecF
Mast cell
4.48
Eosinophil
84.2
8.49
F4/80
CD64
Macrophage
1.13

Figure S6. Gating strategy for flow cytometry.

Cell (surface) markers used to determine the cell identity of B, CD4 T, CD8 T, natural killer (NK) cells, macrophages, monocytes, neutrophils, basophils, eosinophils, mast cells, and dendritic cells, respectively; SSC, side scatter; FSC, forward scatter.

Fig. S7

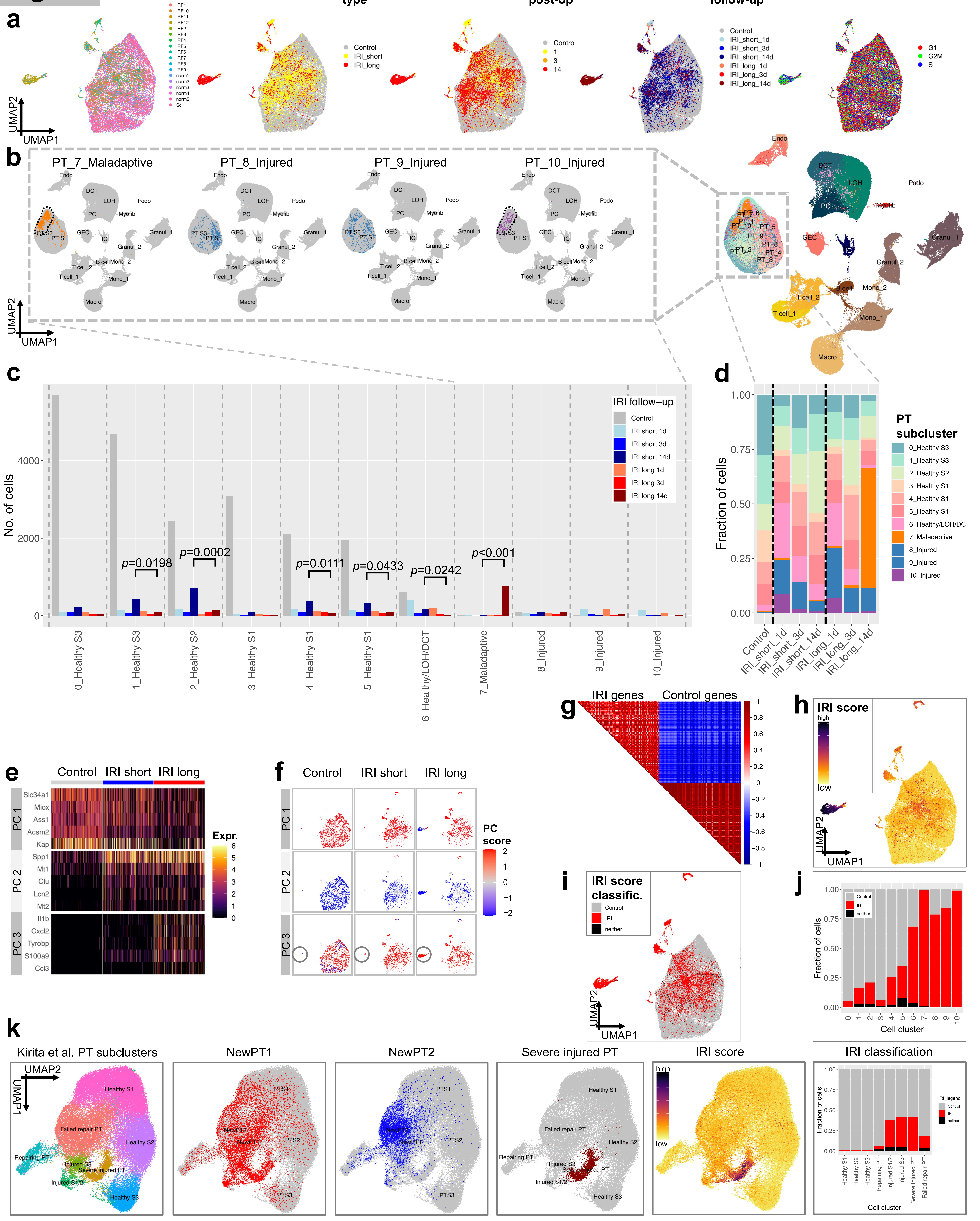
a
Sample
IRI type
Control
IRI_short
IRI_long
Time post-op
Control
1
3
14
IRI follow-up
Control
IRI_short_1d
IRI_short_3d
IRI_short_14d
IRI_long_1d
IRI_long_3d
IRI_long_14d
Cell cycle
G1
G2M
S
UMAP1
UMAP2
b
PT_7_Maladaptive
PT_8_Injured
PT_9_Injured
PT_10_Injured
c
IRI follow-up
Control
IRI short 1d
IRI short 3d
IRI short 14d
IRI long 1d
IRI long 3d
IRI long 14d
No. of cells
p=0.0198
p=0.0002
p=0.0111
p=0.0433
p=0.0242
p<0.001
0_Healthy S3
1_Healthy S3
2_Healthy S2
3_Healthy S1
4_Healthy S1
5_Healthy S1
6_Healthy/LOH/DCT
7_Maladaptive
8_Injured
9_Injured
10_Injured
d
Fraction of cells
PT subcluster
e
Control
IRI short
IRI long
Expr.
f
PC score
g
IRI genes
Control genes
h
IRI score
high
low
i
IRI score classific.
Control
IRI
neither
j
Fraction of cells
Cell cluster
k
Kirita et al. PT subclusters
NewPT1
NewPT2
Severe injured PT
IRI score
IRI classification

Figure S7. PT cell quality control, cell fractions, heterogeneity, and IRI score.

(a) UMAP projection of 28,385 PT cells of n=6 controls, n=6 short IRI and n=6 long IRI samples colored by sample, IRI type (Control, short IRI, long IRI), time post-op (Control, 1d, 3d, 14d), IRI follow-up (Control, IRI short 1d, IRI short 3d, IRI short 14d, IRI long 1d, IRI long 3d, IRI long 14d), cell cycle phase (G1, G2M, S), and GMM cluster (1-5) as inferred by Slingshot analysis of sub-sampled cells.

(b) Right panel shows a UMAP projection of the whole dataset with PT cells colored by PT subcluster as in **Fig. 1f**, as inferred by separate subclustering analysis. Left panels show projections of Maladapative and Injured PT subclusters within the whole dataset.

(c) Number of PT subcluster cells across ischemia dose and time post-IRI (Control, IRI short 1d, IRI short 3d, IRI short 14d, IRI long 1d, IRI long 3d, IRI long 14d). Statistical significance for comparisons between short and long IRI was derived using differential proportion analysis, with a mean error of 0.1 over 100,000 iterations; p values are given for time point comparisons between IRI short and long.

(d) Stacked bar graph showing the fraction of PT subclusters across ischemia dose and time post-IRI (Control, IRI short 1d, IRI short 3d, IRI short 14d, IRI long 1d, IRI long 3d, IRI long 14d).

(e) Heatmap visualizing the expression of top high-loading genes for the first three principal components (PC) based on PC analysis of all PT cells, illustrating recurrent aspects of transcriptional heterogeneity between treatment conditions (Control; IRI short; IRI long).

(f) Corresponding projections of scaled and normalized PC scores across different treatment conditions. The first 3 PCs are visualized. Maladapted PT cells (circled) contributed the most to PC heterogeneity.

(g) Top 100 IRI genes and 100 Control genes used to create an IRI score show very high intra-group correlation and inter-group anticorrelation; scale represents Pearson correlation coefficients.

(h) Calculated IRI scores projected on every single PT cell in UMAP embedded space highlights stark phenotype differences between Healthy and Maladaptive PT subclusters.

(i) IRI score-derived corresponding cell classification into either Control, IRI or neither of the two.

(j) Fraction of cells classified as in **(i)**, stratified by PT subcluster.

(k) Validation of IRI scoring method in PT cells from an external renal IRI dataset (Kirita et al.). Original PT cluster annotations from the external dataset, NewPT1, NewPT2, and Severe injured PT cells are visualized in UMAP space. Note that PT cells with the highest IRI score are located in cluster "Severe injured PT". Right subpanel shows cell classification according to IRI score into either Control, IRI or neither of the two and confirms the presence of IRI-classified cells exclusively in non-healthy cell clusters.

Fig. S8

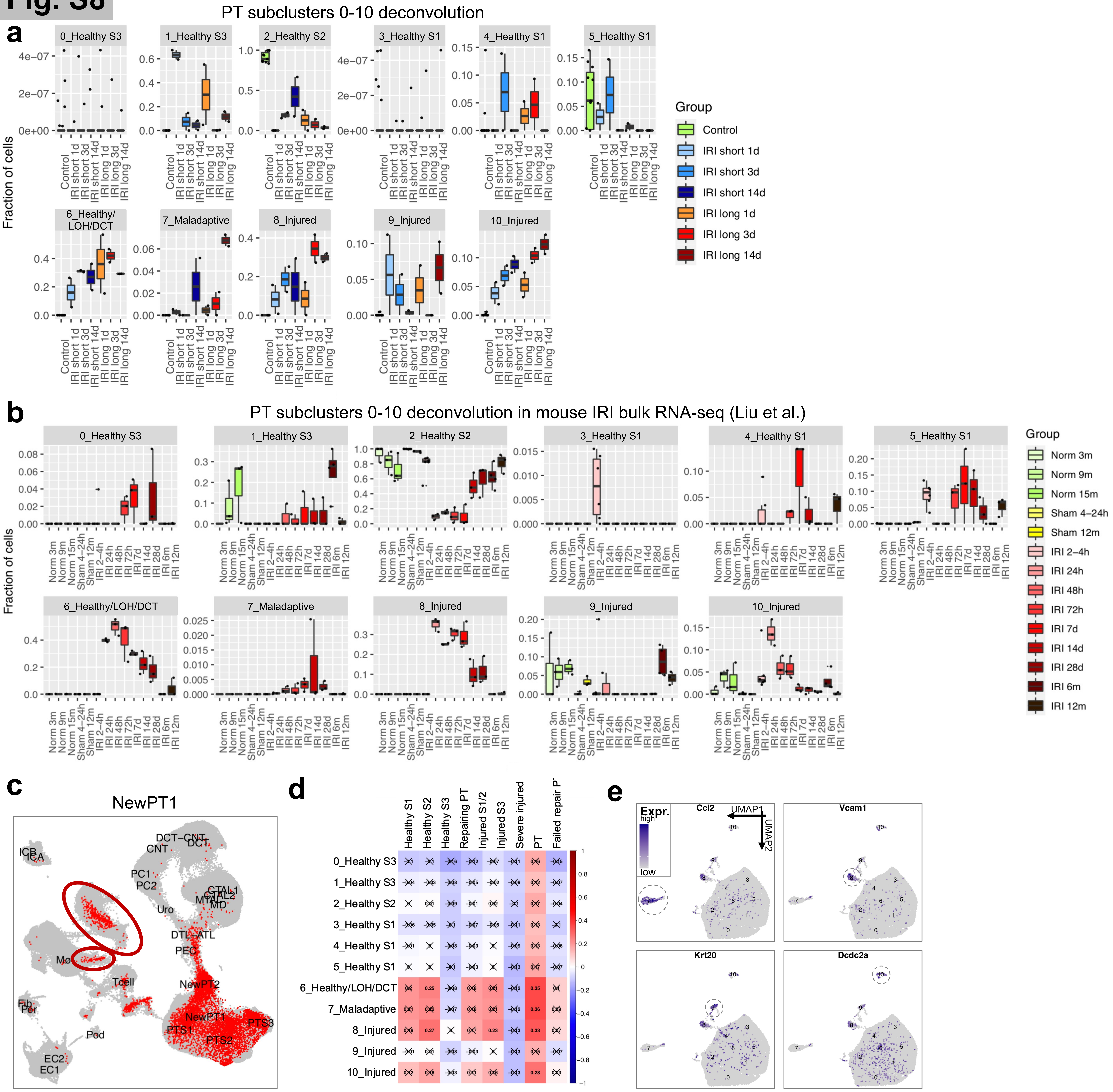

PT subclusters 0-10 deconvolution
a
Fraction of cells
0_Healthy S3
1_Healthy S3
2_Healthy S2
3_Healthy S1
4_Healthy S1
5_Healthy S1
6_Healthy/ LOH/DCT
7_Maladaptive
8_Injured
9_Injured
10_Injured
Control
IRI short 1d
IRI short 3d
IRI short 14d
IRI long 1d
IRI long 3d
IRI long 14d
Group
b
PT subclusters 0-10 deconvolution in mouse IRI bulk RNA-seq (Liu et al.)
6_Healthy/LOH/DCT
Norm 3m
Norm 9m
Norm 15m
Sham 4–24h
Sham 12m
IRI 2–4h
IRI 24h
IRI 48h
IRI 72h
IRI 7d
IRI 14d
IRI 28d
IRI 6m
IRI 12m
c
NewPT1
d
Healthy S1
Healthy S2
Healthy S3
Repairing PT
Injured S1/2
Injured S3
Severe injured
PT
Failed repair PT
e
Expr.
high
low
Ccl2
UMAP1
UMAP2
Vcam1
Krt20
Dcdc2a

Figure S8. PT subclusters bulk RNA-seq deconvolution.

(a) Fraction of Healthy, Injured, and Maladaptive PT subclusters displayed as Tukey box plots after bulk kidney RNA-seq data convolution using the MuSiC pipeline. X axis denotes the different treatment groups (Control, IRI short 1d, IRI short 3d, IRI short 14d, IRI long 1d, IRI long 3d, IRI long 14d).

(b) Same as **(a)** for a mouse kidney IRI dataset from Liu et al. X axis denotes the different treatment groups for Controls (Norm), sham, and IRI kidneys after several different time points.

(c) Cells annotated as "NewPT1" from Kirita et al. are colored red in the joint UMAP embedding. Note significant overlap with Granul_1, Granul_2, and Macro clusters. Cluster labels as in Kirita et al.

(d) Correlation matrix visualizing Pearson correlation coefficients (PCC) of cluster-averaged expression of top 50 DEGs from PT subclusters 0-10 (rows) and PT subclusters from Kirita et al., columns. Non-significant PCC values are crossed out.

(e) Feature plots of selected marker genes from Kirita et al. PT subclusters projected onto UMAP space as in **Fig. 2a**.

Fig. S9

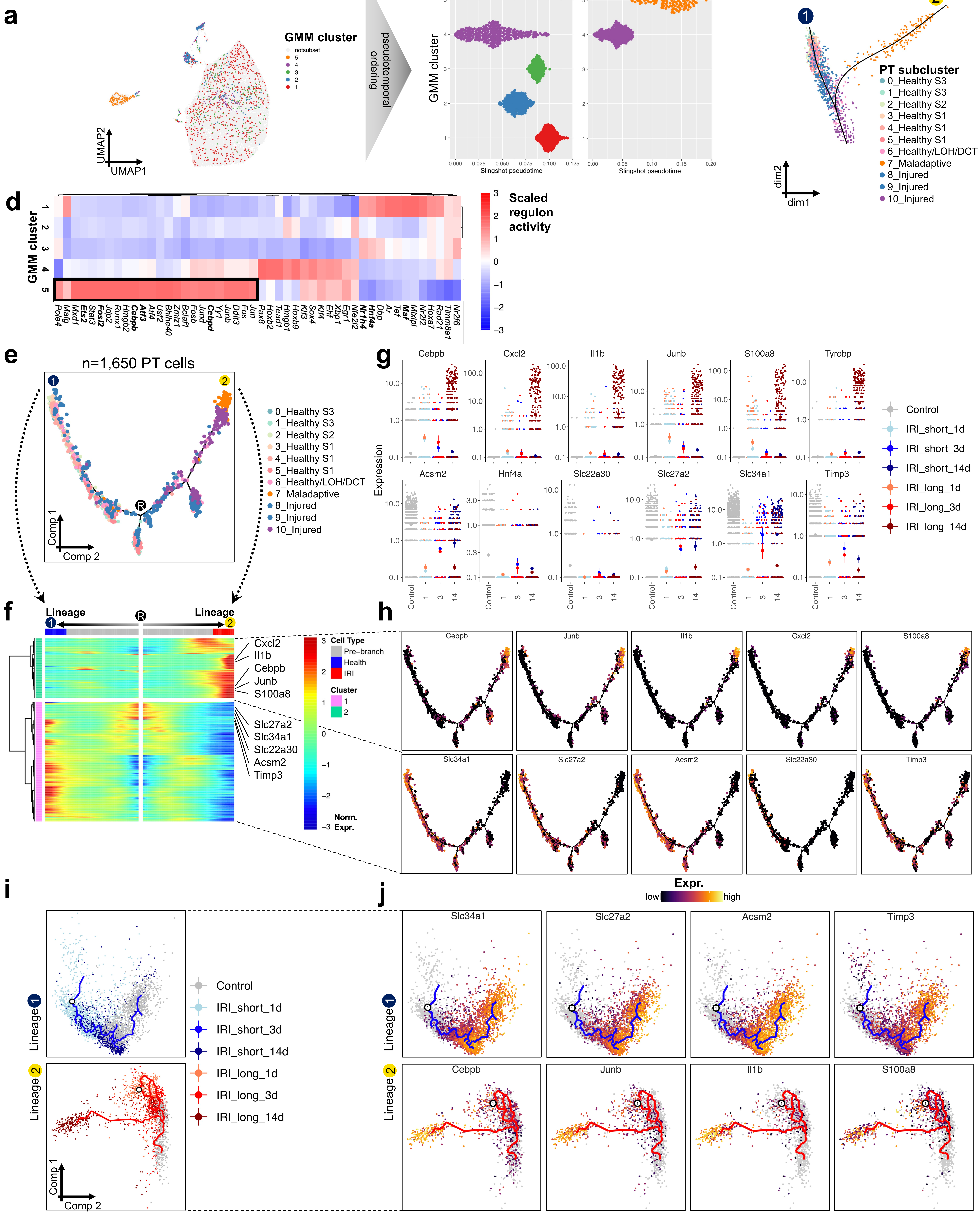

a
GMM cluster
notsubset
5
4
3
2
1
UMAP2
UMAP1
pseudotemporal ordering
b
Lineage 1
Lineage 2
GMM cluster
Slingshot pseudotime
c
PT subcluster
0_Healthy S3
1_Healthy S3
2_Healthy S2
3_Healthy S1
4_Healthy S1
5_Healthy S1
6_Healthy/LOH/DCT
7_Maladaptive
8_Injured
9_Injured
10_Injured
dim2
dim1
d
GMM cluster
Scaled regulon activity
Pole4 Mafg Mxd1 Ets2 Stat3 Fosl2 Runx1 Jdp2 Hmgb2 Cebpb Atf3 Atf4 Usf2 Bhlhe40 Zmiz1 Bclaf1 Fosb Jund Cebpd Yy1 Ddit3 Junb Fos Jun Pax8 Hoxb2 Tead1 Hmgb1 Hoxb9 Klf3 Sox4 Klf4 Ehf Xbp1 Egr1 Nfe2l2 Nr1h4 Hnf4a Dbp Ar Tef Maf Mlxipl Nr2f2 Hoxa7 Rad21 Timm8a1 Nr2f6
e
n=1,650 PT cells
Comp 1
Comp 2
f
Lineage 1
Lineage 2
Cxcl2
Il1b
Cebpb
Junb
S100a8
Slc27a2
Slc34a1
Slc22a30
Acsm2
Timp3
Cell Type
Pre-branch
Health
IRI
Cluster
Norm. Expr.
g
Cebpb
Cxcl2
Il1b
Junb
S100a8
Tyrobp
Acsm2
Hnf4a
Slc22a30
Slc27a2
Slc34a1
Timp3
Expression
Control
IRI_short_1d
IRI_short_3d
IRI_short_14d
IRI_long_1d
IRI_long_3d
IRI_long_14d
h
Cebpb
Junb
Il1b
Cxcl2
S100a8
Slc34a1
Slc27a2
Acsm2
Slc22a30
Timp3
i
Lineage 1
Lineage 2
Control
IRI_short_1d
IRI_short_3d
IRI_short_14d
IRI_long_1d
IRI_long_3d
IRI_long_14d
Comp 1
Comp 2
j
Expr.
low
high
Slc34a1
Slc27a2
Acsm2
Timp3
Cebpb
Junb
Il1b
S100a8

Figure S9. PT injury trajectories and differential driver patterns of adaptive and maladaptive PT cell repair.

(a-b) 1,650 randomly sampled PT cells **(a)** are ordered by Slingshot pseudotime along lineages 1 (dark blue label) and 2 (yellow label) **(b)**. Clusters correspond to Gaussian mixed modeling (GMM) clustering, as inferred by Slingshot.

(c) Dimensional reduction representation of Slingshot-derived cell trajectories of PT cells, coloring corresponds to PT subclusters as in **Figs. 2a-c**.

(d) Heatmap of GMM cluster-specific SCENIC-inferred scaled regulon activity. Rows represent GMM clusters, columns represent regulons, as inferred by Slingshot in **Fig. 3a**.

(e) Monocle2-derived trajectory of the same PT cell selection, confirming lineages 1 and 2. R denotes the root node where branching of lineages 1 and 2 occurs.

(f) Heatmap visualizing pseudotiome-dependent gene expression along lineages 1 and 2, respectively. R denotes the root node where branching of lineages 1 and 2 occurs, as shown in **(e)**.

(g) Gene expression of DEGs from **(f)** colored by experimental sample group stratified by IRI degree and time post-ischemia.

(h) Corresponding Monocle2 visualization of gene expression in reduced dimension space.

(i) Monocle3-derived trajectories of lineages 1 and 2 of the same PT cell selection, coloring as per experimental group.

(j) Corresponding Monocle3 visualization of gene expression in reduced dimension space.

Fig. S10

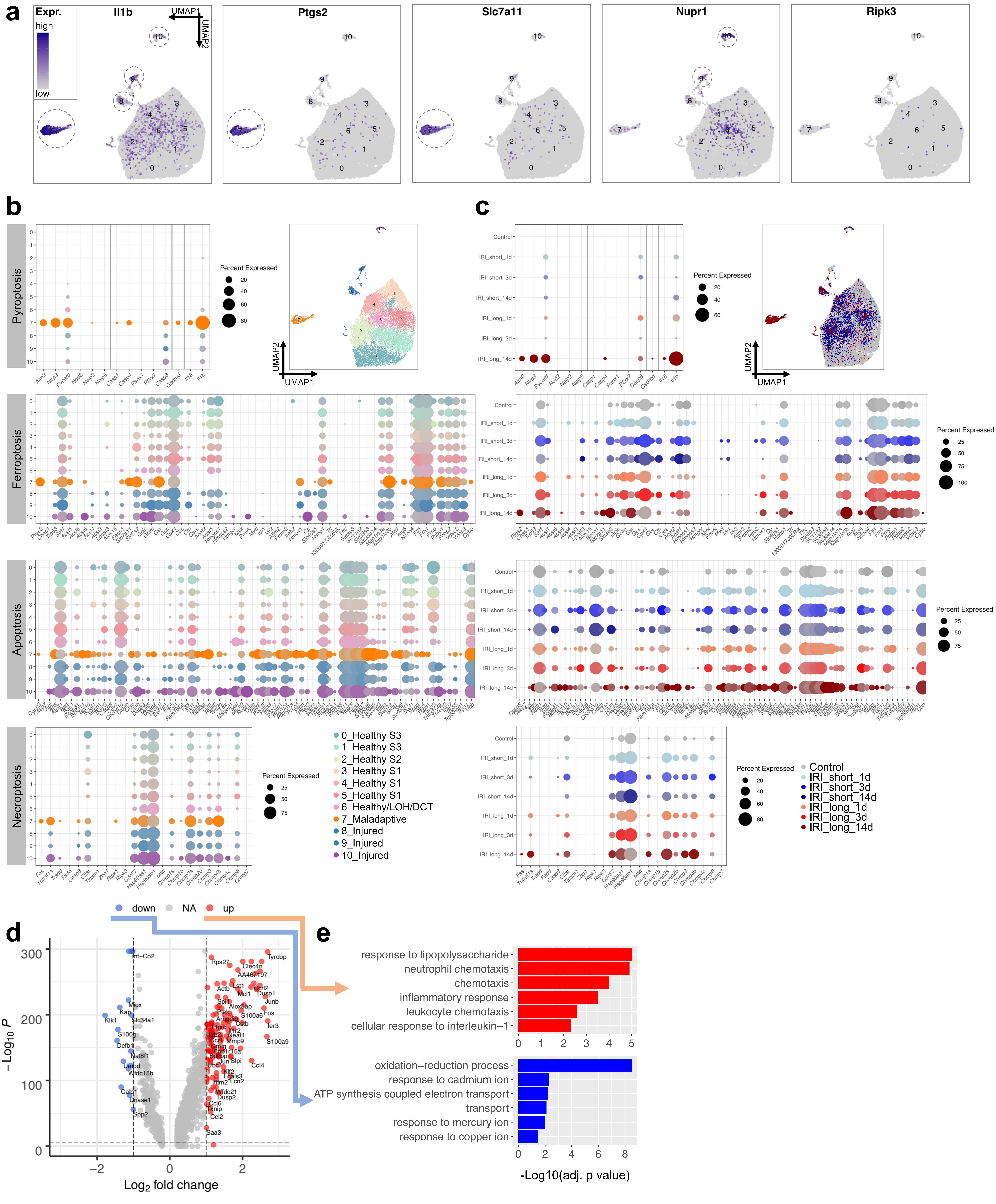

a
Expr.
high
low
Il1b
UMAP1
UMAP2
Ptgs2
Slc7a11
Nupr1
Ripk3
b
Pyroptosis
Ferroptosis
Apoptosis
Necroptosis
Percent Expressed
UMAP2
UMAP1
0_Healthy S3
1_Healthy S3
2_Healthy S2
3_Healthy S1
4_Healthy S1
5_Healthy S1
6_Healthy/LOH/DCT
7_Maladaptive
8_Injured
9_Injured
10_Injured
c
Control
IRI_short_1d
IRI_short_3d
IRI_short_14d
IRI_long_1d
IRI_long_3d
IRI_long_14d
d
down
NA
up
−Log10 P
Log2 fold change
e
response to lipopolysaccharide
neutrophil chemotaxis
chemotaxis
inflammatory response
leukocyte chemotaxis
cellular response to interleukin−1
oxidation−reduction process
response to cadmium ion
ATP synthesis coupled electron transport
transport
response to mercury ion
response to copper ion
-Log10(adj. p value)

Figure S10. Sustained regulated cell death signatures in PT cells after long IRI.

(a) Feature plots of selected genes from **Fig. 4b** projected onto UMAP space as in **Fig. 2a**.

(b-c) Dot plots showing comprehensive marker gene expression in PT cells for regulated cell death necrosis pathways pyroptosis, ferroptosis, apoptosis, and necroptosis. Cells are stratified by PT subclusters **(b)** or ischemia dose and time post-IRI **(c)**. Dot size denotes percentage of cells expressing the marker.

(d) Volcano plot of differentially expressed genes (DEGs) comparing PT cells from samples 14d after short and long IRI, respectively. Genes upregulated in IRI long compared to IRI short samples are colored red, downregulated genes are colored blue. X axis indicates log_2-fold change (Model-based Analysis of Single-cell Transcriptomics; MAST) and y axis indicates statistical significance-adjusted p = $-log_{10}$.

(e) Corresponding Gene Ontology (GO) analysis showing statistically significantly enriched GO terms (over-representation analysis with Benjamini-Hochberg FDR method) corresponding to up- and downregulated DEGs from **(d)**.

Fig. S11

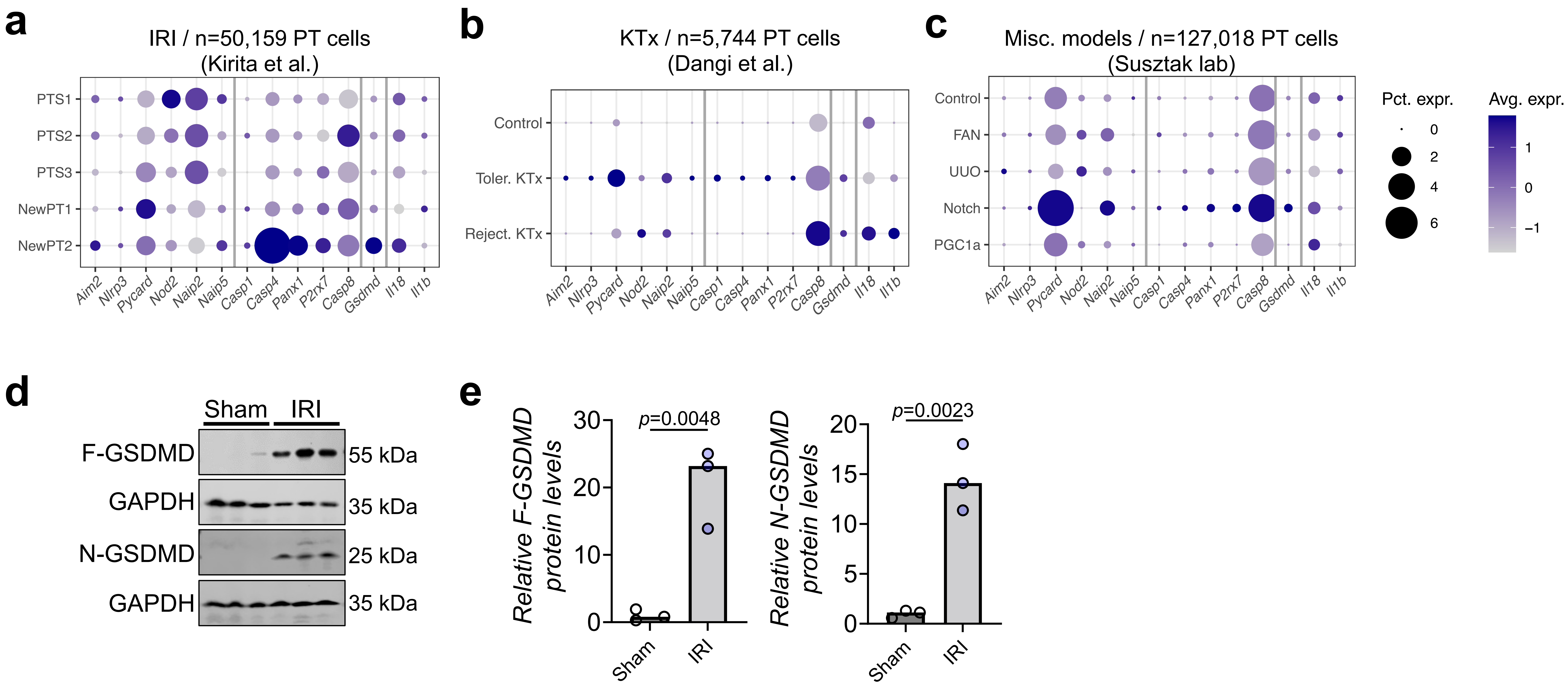
a
IRI / n=50,159 PT cells
(Kirita et al.)
PTS1
PTS2
PTS3
NewPT1
NewPT2
b
KTx / n=5,744 PT cells
(Dangi et al.)
Control
Toler. KTx
Reject. KTx
c
Misc. models / n=127,018 PT cells
(Susztak lab)
Control
FAN
UUO
Notch
PGC1a
Aim2
Nlrp3
Pycard
Nod2
Naip2
Naip5
Casp1
Casp4
Panx1
P2rx7
Casp8
Gsdmd
Il18
Il1b
Pct. expr.
0
2
4
6
Avg. expr.
1
0
−1
d
Sham
IRI
F-GSDMD
55 kDa
GAPDH
35 kDa
N-GSDMD
25 kDa
GAPDH
35 kDa
e
Relative F-GSDMD protein levels
p=0.0048
Relative N-GSDMD protein levels
p=0.0023
Sham
IRI

Figure S11. Pyroptosis gene signature in PT cells of other kidney scRNA-seq datasets.

(a-c) Dot plots showing pyroptosis marker gene expression in PT cells of 2 external datasets (Kirita et al. and Dangi et al.) and several mouse models of our own lab, respectively. Dot size denotes percentage of cells expressing the marker, color scale represents average gene expression values. FAN, folic acid nephropathy; Notch; conditional tubular overexpression of Notch mouse model; UUO, unilateral ureteric obstruction; KTx, kidney transplant; reject., rejecting; toler., tolerated.

(d) Western blot for full length (GSDMD-F) and cleaved GSDMD (GSDMD-N) in full kidney lysates. GAPDH was used as loading control. IRI, long ischemia reperfusion injury.

(e) Corresponding quantification of Western blot in **(d)**; n=3 independent experiments, data shown are from n=1 experiment with n=3 biological replicates per group; p values are given for Wilcoxon-Mann test.

Fig. S12

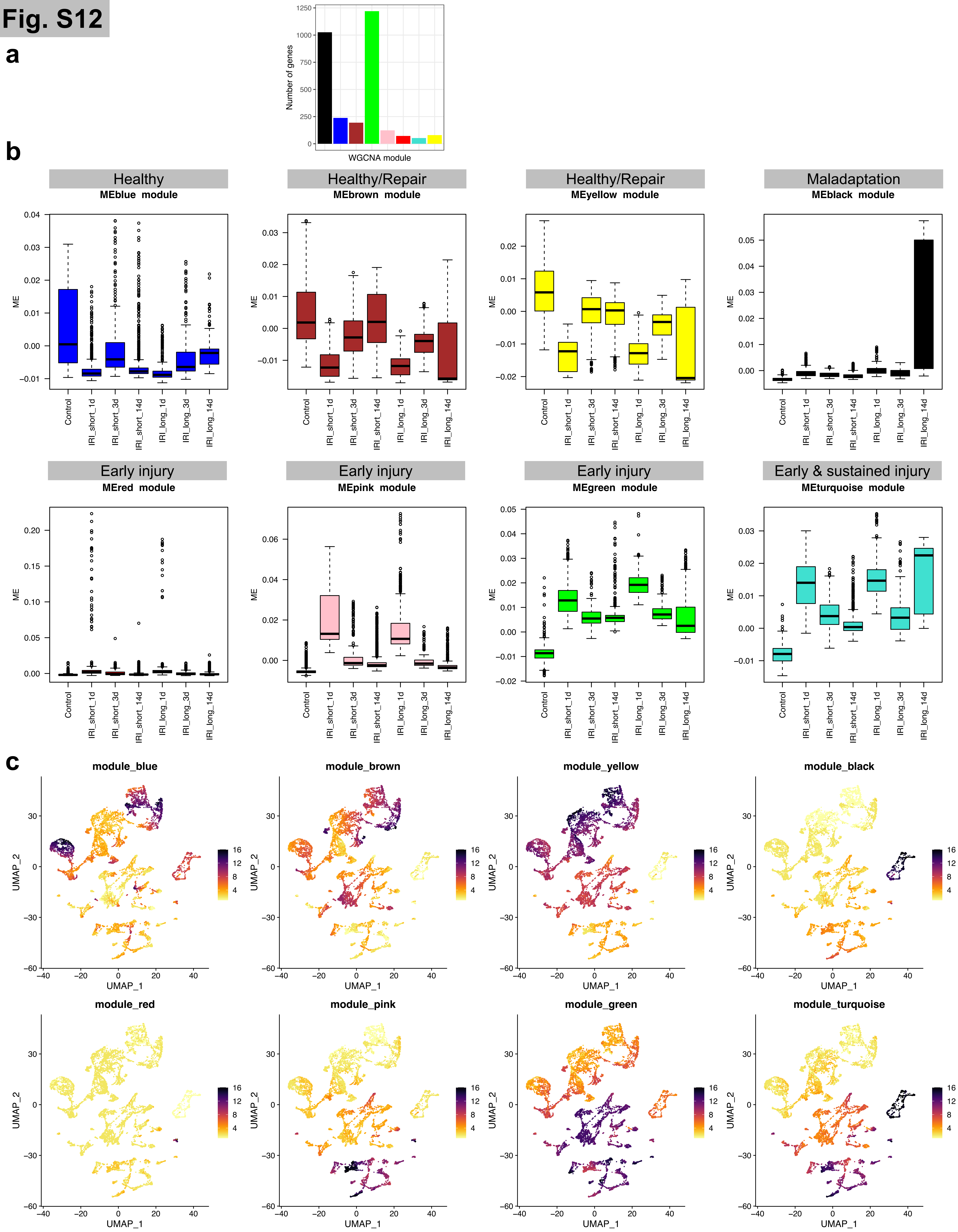
a
Number of genes
WGCNA module
1250
1000
750
500
250
0
b
Healthy
MEblue module
Healthy/Repair
MEbrown module
Healthy/Repair
MEyellow module
Maladaptation
MEblack module
Early injury
MEred module
Early injury
MEpink module
Early injury
MEgreen module
Early & sustained injury
MEturquoise module
ME
Control
IRI_short_1d
IRI_short_3d
IRI_short_14d
IRI_long_1d
IRI_long_3d
IRI_long_14d
c
module_blue
module_brown
module_yellow
module_black
module_red
module_pink
module_green
module_turquoise
UMAP_1
UMAP_2
16
12
8
4

Figure S12. WGCNA analysis in PT metacells.

(a) Bar graph depicting the number of genes in WGCNA-derived modules.

(b) Module eigengene (ME) scores of WGCNA modules displayed as Tukey box plots (outliers denoted as dots outside box plot whiskers) by IRI degree and time post-ischemia (n=8,777 metacells).

(c) kME values of individual WGCNA modules visualized in UMAP space.

Fig. S13

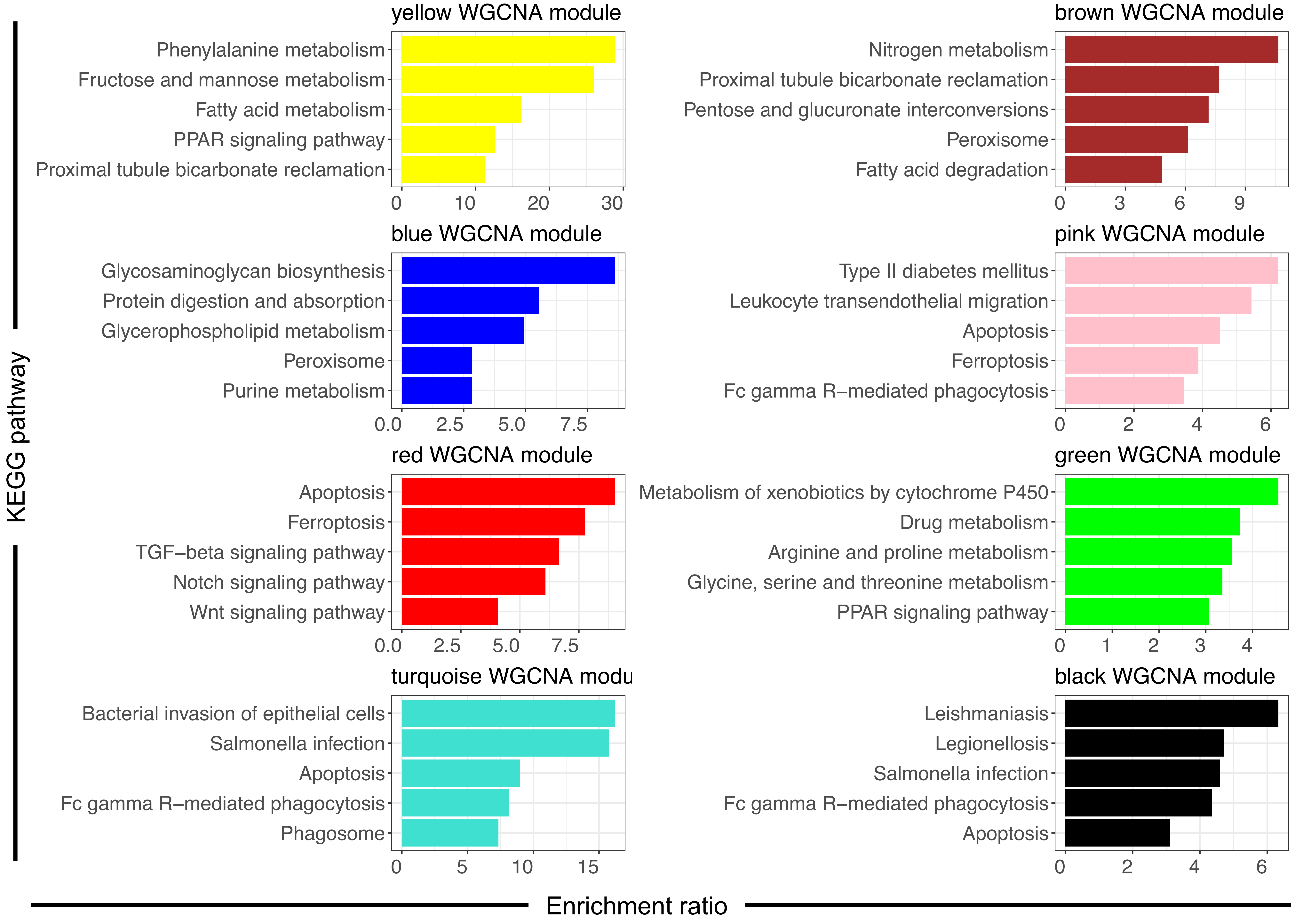

yellow WGCNA module
Phenylalanine metabolism
Fructose and mannose metabolism
Fatty acid metabolism
PPAR signaling pathway
Proximal tubule bicarbonate reclamation
0 10 20 30
brown WGCNA module
Nitrogen metabolism
Proximal tubule bicarbonate reclamation
Pentose and glucuronate interconversions
Peroxisome
Fatty acid degradation
0 3 6 9
blue WGCNA module
Glycosaminoglycan biosynthesis
Protein digestion and absorption
Glycerophospholipid metabolism
Peroxisome
Purine metabolism
0.0 2.5 5.0 7.5
pink WGCNA module
Type II diabetes mellitus
Leukocyte transendothelial migration
Apoptosis
Ferroptosis
Fc gamma R−mediated phagocytosis
0 2 4 6
red WGCNA module
Apoptosis
Ferroptosis
TGF−beta signaling pathway
Notch signaling pathway
Wnt signaling pathway
0.0 2.5 5.0 7.5
green WGCNA module
Metabolism of xenobiotics by cytochrome P450
Drug metabolism
Arginine and proline metabolism
Glycine, serine and threonine metabolism
PPAR signaling pathway
0 1 2 3 4
turquoise WGCNA modu
Bacterial invasion of epithelial cells
Salmonella infection
Apoptosis
Fc gamma R−mediated phagocytosis
Phagosome
0 5 10 15
black WGCNA module
Leishmaniasis
Legionellosis
Salmonella infection
Fc gamma R−mediated phagocytosis
Apoptosis
0 2 4 6
KEGG pathway
Enrichment ratio

Figure S13. KEGG pathway analysis for WGCNA modules.

Enrichment ratios of top enriched KEGG pathway terms, derived by overrepresentation analysis of WGCNA module genes in proximal tubule metacells. The graph is split into subpanels by WGCNA module.

Fig. S14

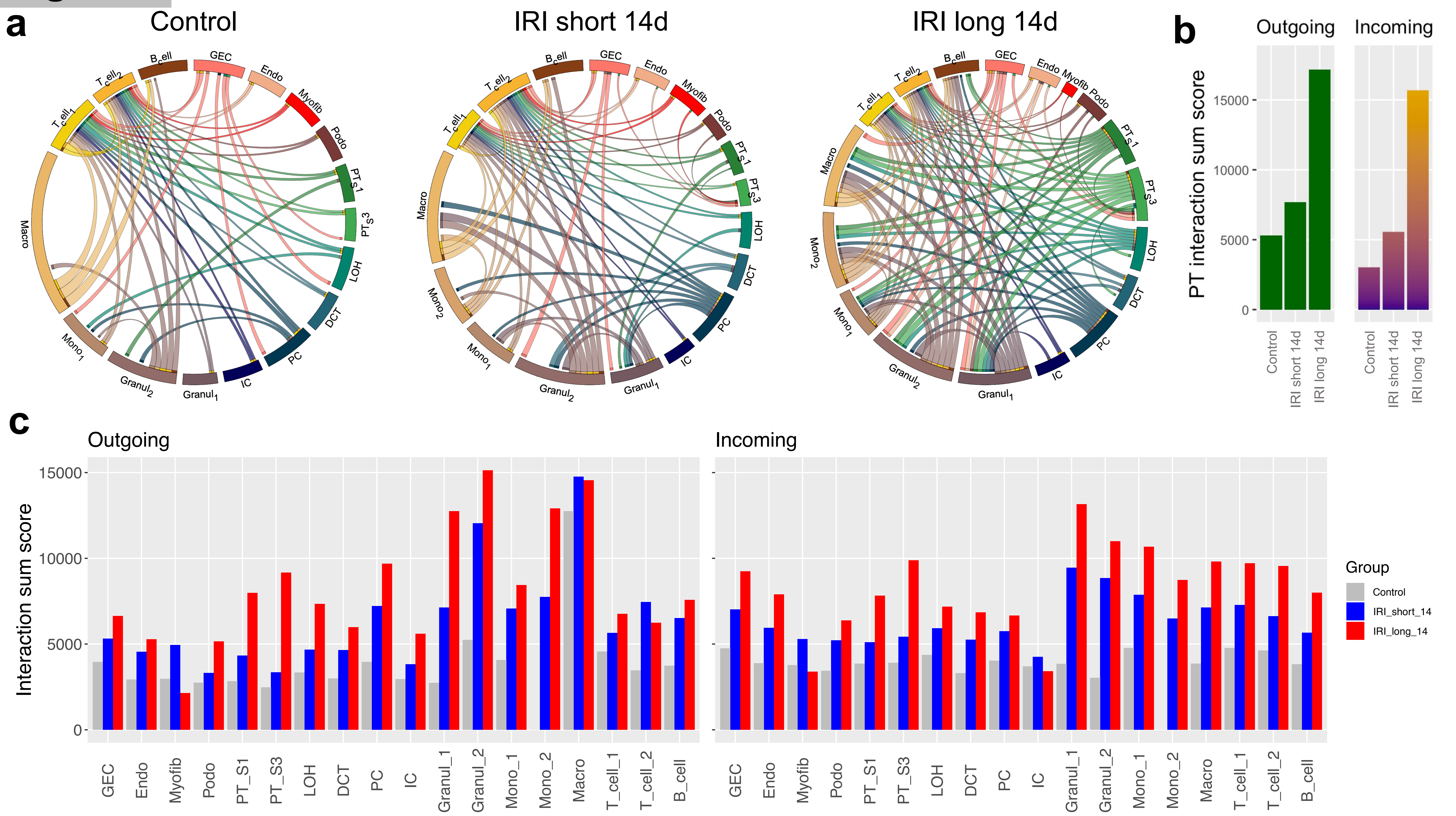
a
Control
IRI short 14d
IRI long 14d
B_cell
GEC
Endo
Myofib
Podo
PT_S1
PT_S3
LOH
DCT
PC
IC
Granul_1
Granul_2
Mono_1
Mono_2
Macro
T_cell_1
T_cell_2
b
Outgoing
Incoming
PT interaction sum score
15000
10000
5000
0
Control
IRI short 14d
IRI long 14d
c
Outgoing
Incoming
Interaction sum score
Group
Control
IRI_short_14
IRI_long_14

Figure S14. Cell-cell interaction of kidney cells in health, adaptive repair, and maladaptive repair.

(a) Circos plots visualizing the degree of cell-cell interactions as inferred by CellPhoneDB between healthy (Control), adapted (IRI short 14d), and maladapted (IRI long 14d) kidney cells. Width of connecting lines is proportional to the strength of interaction, as measured by combined means of ligands and respective receptors by CellPhoneDB. For clarity only the top 50% of interactions are visualized.

(b) Bar graphs showing the sum score of outgoing and incoming connections of proximal tubule (PT) cells across treatment conditions (Control; IRI short 14d; IRI long 14d).

(c) Bar graphs showing the sum score of outgoing and incoming connections of all cell types across treatment conditions (Control; IRI short 14d; IRI long 14d).

Fig. S15

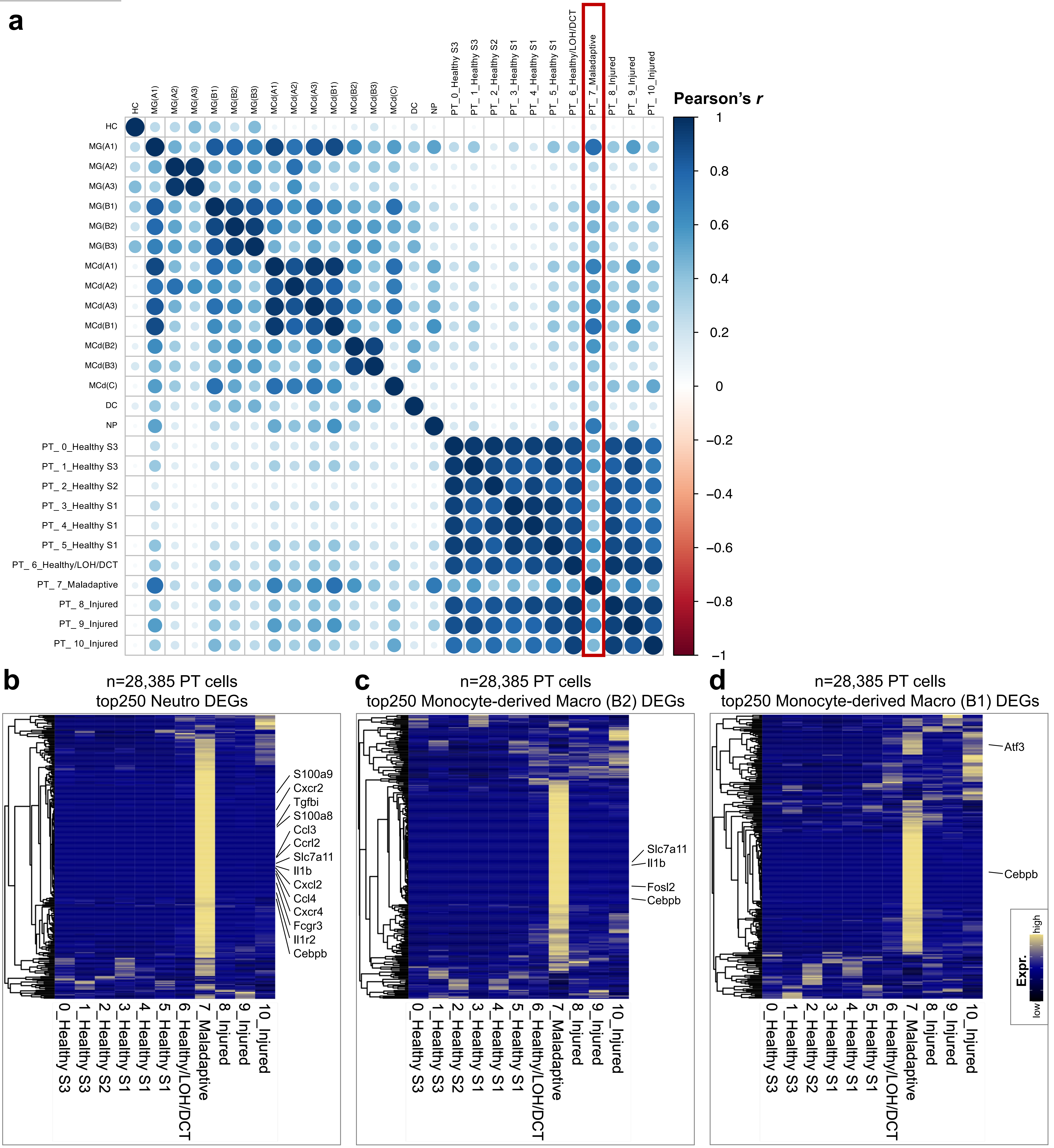
a
Pearson's r
1
0.8
0.6
0.4
0.2
0
−0.2
−0.4
−0.6
−0.8
−1
HC
MG(A1)
MG(A2)
MG(A3)
MG(B1)
MG(B2)
MG(B3)
MCd(A1)
MCd(A2)
MCd(A3)
MCd(B1)
MCd(B2)
MCd(B3)
MCd(C)
DC
NP
PT_ 0_Healthy S3
PT_ 1_Healthy S3
PT_ 2_Healthy S2
PT_ 3_Healthy S1
PT_ 4_Healthy S1
PT_ 5_Healthy S1
PT_ 6_Healthy/LOH/DCT
PT_ 7_Maladaptive
PT_ 8_Injured
PT_ 9_Injured
PT_ 10_Injured
b
n=28,385 PT cells
top250 Neutro DEGs
S100a9
Cxcr2
Tgfbi
S100a8
Ccl3
Ccrl2
Slc7a11
Il1b
Cxcl2
Ccl4
Cxcr4
Fcgr3
Il1r2
Cebpb
c
n=28,385 PT cells
top250 Monocyte-derived Macro (B2) DEGs
Slc7a11
Il1b
Fosl2
Cebpb
d
n=28,385 PT cells
top250 Monocyte-derived Macro (B1) DEGs
Atf3
Cebpb
Expr.
high
low
0_Healthy S3
1_Healthy S3
2_Healthy S2
3_Healthy S1
4_Healthy S1
5_Healthy S1
6_Healthy/LOH/DCT
7_Maladaptive
8_Injured
9_Injured
10_Injured

Figure S15. Validation of myeloid phenotype of maladaptive PT cells.

(a) Correlation matrix visualizing Pearson correlation coefficients between average expressions of clusters from an external dataset of immune cells in spinal cord injury (Hamel et al.) and our PT subclusters. PT_7_Maladaptive gene signature correlates best with neutrophils (NP), monocyte-derived macrophages (MCd) B1 and B2, corresponding to different pseudotime points on a trajectory characterized by cell death.

(b-d) Heatmaps visualizing gene expression in 11 subclusters of 28,385 PT cells of marker genes from an external dataset of immune cells in spinal cord injury (Hamel et al.). **(b)** depicts top 250 marker genes for neutrophils, **(c)** and **(d)** for monocyte-derived macrophages at different pseudotime points on a trajectory characterized by cell death.

Fig. S16

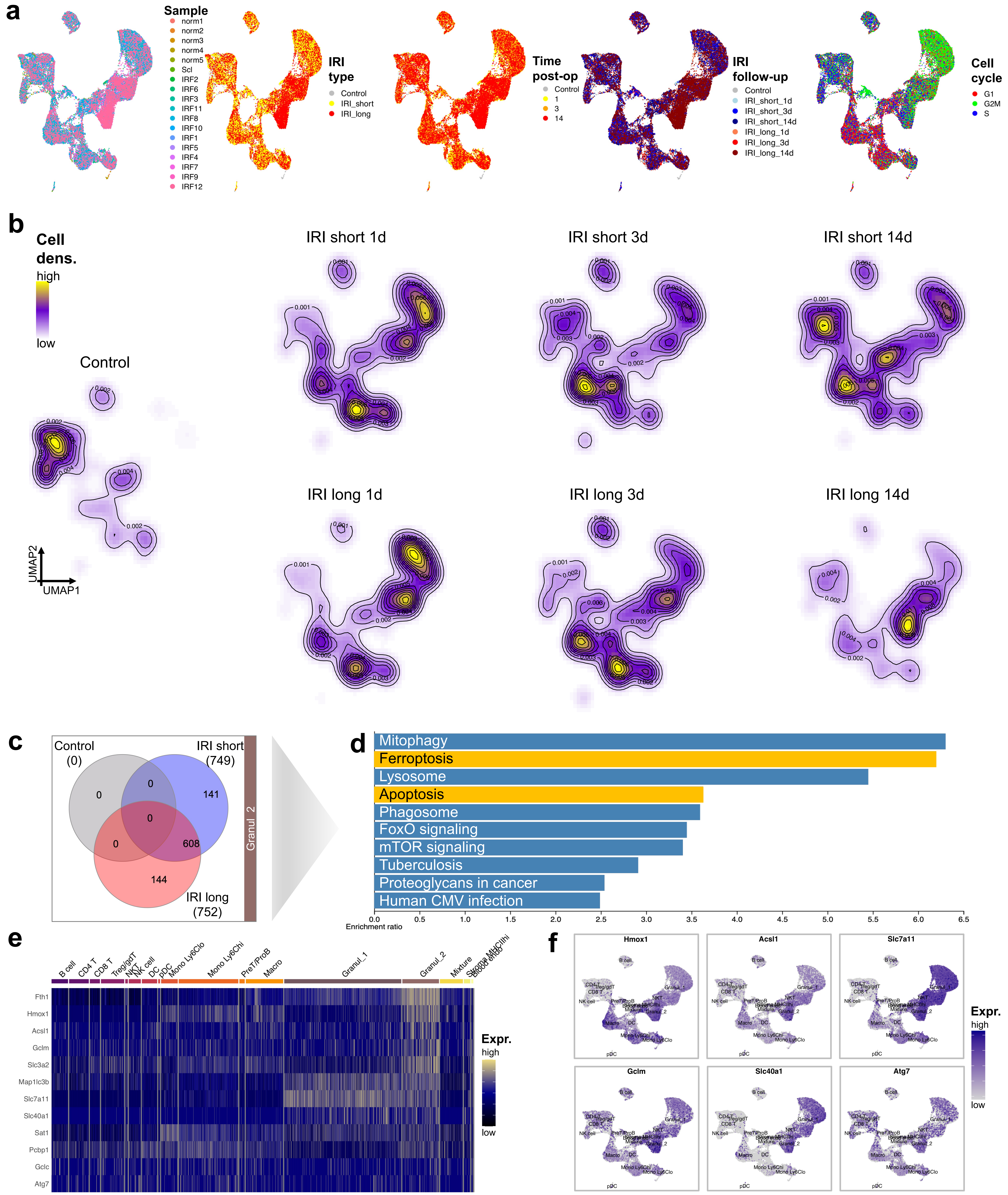
a
Sample
norm1
norm2
norm3
norm4
norm5
Scl
IRF2
IRF6
IRF3
IRF11
IRF8
IRF10
IRF1
IRF5
IRF4
IRF7
IRF9
IRF12
IRI type
Control
IRI_short
IRI_long
Time post-op
Control
1
3
14
IRI follow-up
Control
IRI_short_1d
IRI_short_3d
IRI_short_14d
IRI_long_1d
IRI_long_3d
IRI_long_14d
Cell cycle
G1
G2M
S
b
Cell dens.
high
low
Control
IRI short 1d
IRI short 3d
IRI short 14d
IRI long 1d
IRI long 3d
IRI long 14d
UMAP2
UMAP1
c
Control (0)
IRI short (749)
IRI long (752)
0
0
141
0
0
608
144
Granul_2
d
Mitophagy
Ferroptosis
Lysosome
Apoptosis
Phagosome
FoxO signaling
mTOR signaling
Tuberculosis
Proteoglycans in cancer
Human CMV infection
0.0
0.5
1.0
1.5
2.0
2.5
3.0
3.5
4.0
4.5
5.0
5.5
6.0
6.5
Enrichment ratio
e
B cell
CD4 T
CD8 T
Treg/gdT
NKT
NK cell
DC
pDC
Mono Ly6Clo
Mono Ly6Chi
PreT/ProB
Macro
Granul_1
Granul_2
Mixture
Stroma MHCIIhi
Blood endo
Fth1
Hmox1
Acsl1
Gclm
Slc3a2
Map1lc3b
Slc7a11
Slc40a1
Sat1
Pcbp1
Gclc
Atg7
Expr.
high
low
f
Hmox1
Acsl1
Slc7a11
Gclm
Slc40a1
Atg7
Expr.
high
low

Figure S16. Transcriptional atlas of immune cells in acutely injured kidneys.

(a) UMAP projection of 45,327 immune cells of n=6 controls, n=6 short IRI and n=6 long IRI samples colored by sample, IRI type (Control, short IRI, long IRI), time post-op (Control, 1d, 3d, 14d), IRI follow-up (Control, IRI short 1d, IRI short 3d, IRI short 14d, IRI long 1d, IRI long 3d, IRI long 14d), and cell cycle phase (G1, G2M, S).

(b) Density plots visualizing changes in cell type proportions between different treatment groups.

(c) Venn diagram visualizing the number of DEGs from Granul_2 cluster, respectively, and their overlap according to treatment group (Control; IRI short; IRI long). Healthy Control samples did not contribute a single cell to Granul_2 cluster.

(d) Gene ontology (GO) terms analysis for DEGs from Granul_2 cluster in **(b)** yielded high enrichment ratios for cell death pathways such as ferroptosis and apoptosis, respectively.

(e) Heatmap showing the expression of genes known to be involved in ferroptosis by immune cell type. Note the enrichment of myeloid clusters, especially Granul_2, for high expressing cells.

(f) Corresponding feature plots visualizing the expression of select examples from **(d)** in the joint UMAP embedding space.

Fig. S17

a

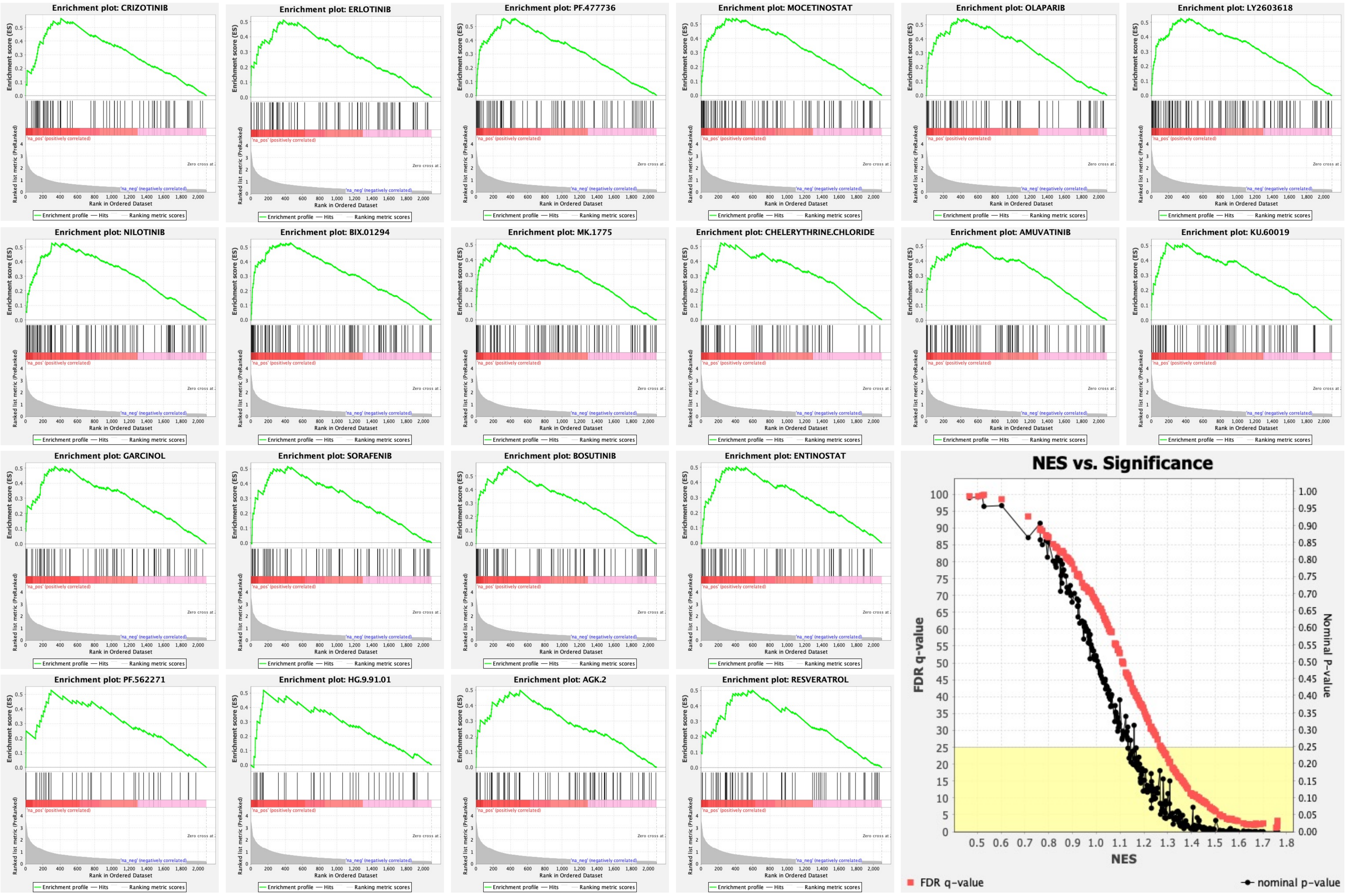

Enrichment plot: CRIZOTINIB
Enrichment plot: ERLOTINIB
Enrichment plot: PF.477736
Enrichment plot: MOCETINOSTAT
Enrichment plot: OLAPARIB
Enrichment plot: LY2603618
Enrichment plot: NILOTINIB
Enrichment plot: BIX.01294
Enrichment plot: MK.1775
Enrichment plot: CHELERYTHRINE.CHLORIDE
Enrichment plot: AMUVATINIB
Enrichment plot: KU.60019
Enrichment plot: GARCINOL
Enrichment plot: SORAFENIB
Enrichment plot: BOSUTINIB
Enrichment plot: ENTINOSTAT
Enrichment plot: PF.562271
Enrichment plot: HG.9.91.01
Enrichment plot: AGK.2
Enrichment plot: RESVERATROL
NES vs. Significance
FDR q-value
Nominal P-value
NES
FDR q-value
nominal p-value

b

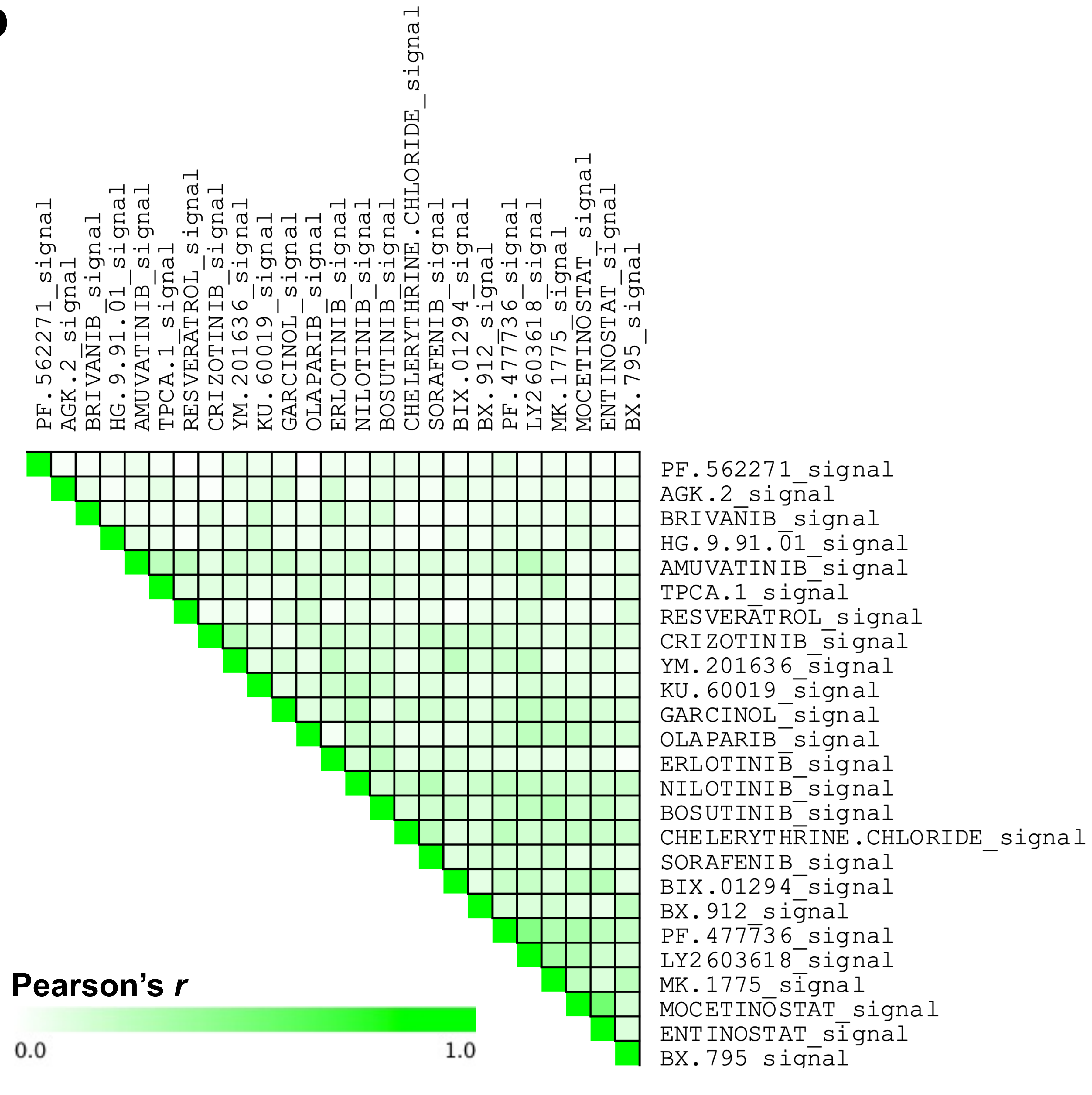

PF.562271_signal
AGK.2_signal
BRIVANIB_signal
HG.9.91.01_signal
AMUVATINIB_signal
TPCA.1_signal
RESVERATROL_signal
CRIZOTINIB_signal
YM.201636_signal
KU.60019_signal
GARCINOL_signal
OLAPARIB_signal
ERLOTINIB_signal
NILOTINIB_signal
BOSUTINIB_signal
CHELERYTHRINE.CHLORIDE_signal
SORAFENIB_signal
BIX.01294_signal
BX.912_signal
PF.477736_signal
LY2603618_signal
MK.1775_signal
MOCETINOSTAT_signal
ENTINOSTAT_signal
BX.795_signal
Pearson's r
0.0
1.0

Figure S17. Gene set enrichment analysis (GSEA) of maladaptive DEGs within drug response lists.

(a) GSEA plots of top 20 drug response patterns overlapping with pre-ranked maladaptive DEGs (Methods). Large subpanel x axis denotes normalized enrichment score (NES), y axis denotes FDR q-value of all tested drug response patterns.

(b) Correlation matrix of top 25 positively enriched drug response patterns.

Fig. S18

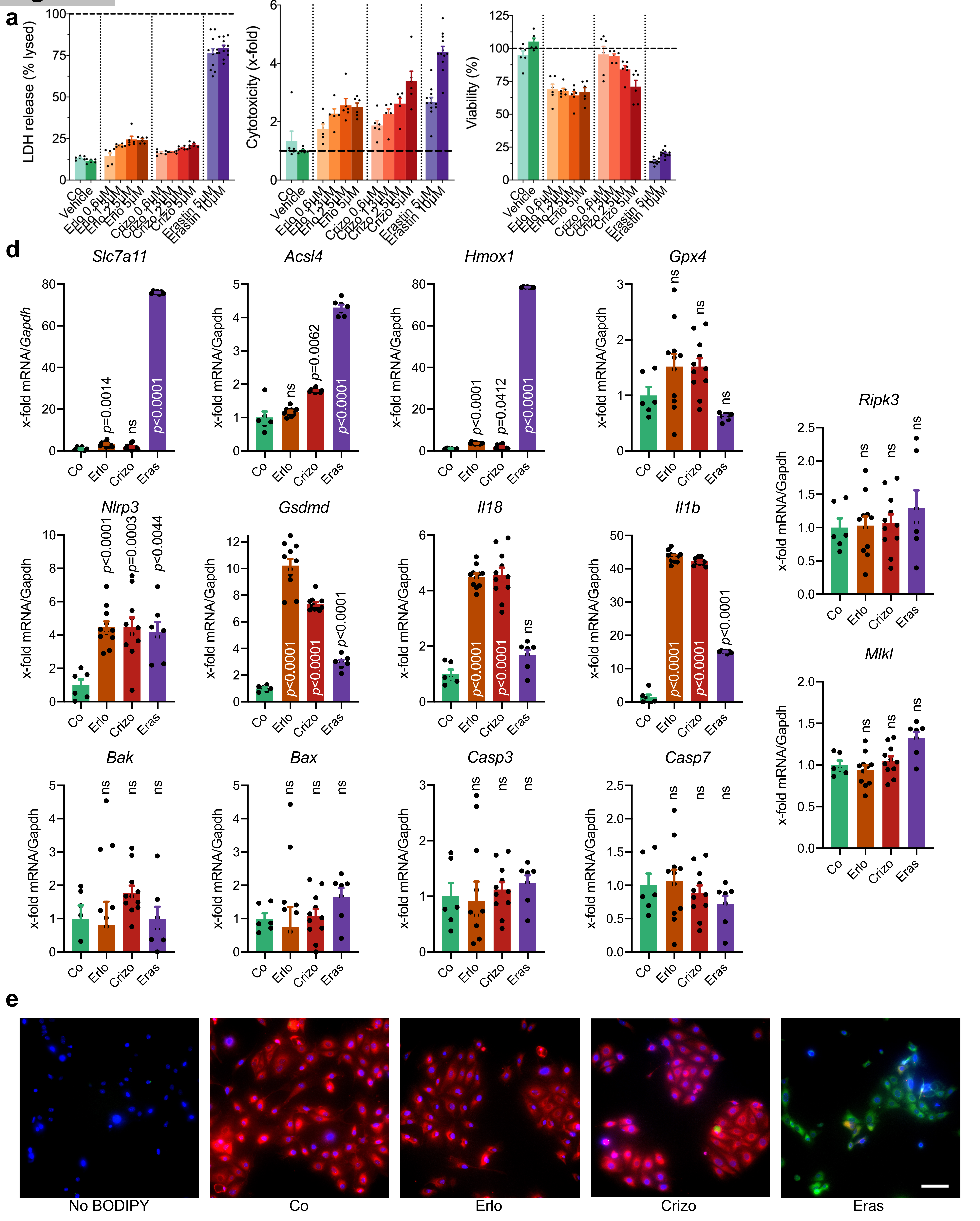

a
LDH release (% lysed)
b
Cytotoxicity (x-fold)
c
Viability (%)
Co
Vehicle
Erlo 0.6µM
Erlo 1.3µM
Erlo 2.5µM
Erlo 5µM
Crizo 0.6µM
Crizo 1.3µM
Crizo 2.5µM
Crizo 5µM
Erastin 5µM
Erastin 10µM
d
Slc7a11
Acsl4
Hmox1
Gpx4
Ripk3
Nlrp3
Gsdmd
Il18
Il1b
Mlkl
Bak
Bax
Casp3
Casp7
x-fold mRNA/Gapdh
Co
Erlo
Crizo
Eras
p=0.0014
ns
p<0.0001
p=0.0062
p<0.0001
p=0.0412
p=0.0003
p=0.0044
e
No BODIPY
Co
Erlo
Crizo
Eras

Figure S18. Erlotinib and crizotinib induced pyroptosis, not ferroptosis, apoptosis, or necroptosis in primary mouse tubular epithelial cell culture *in vitro*.

(a-c) Lactate dehydrogenase (LDH) release, cytotoxicity, and viability of primary mouse tubular epithelial cells treated for 24 h with culture media only (control, Co), vehicle, erlotinib, crizotinib, and ferroptosis inducer erastin, respectively. LDH release is given as percentage of maximum LDH release in fully lysed cells, cytotoxicity and viability are normalized to Co and vehicle groups; n=2 independent experiments; means ± SEM. 5 µM concentrations were hence used for further experiments.

(d) Relative mRNA levels of ferroptosis (*Slc7a11, Acsl4, Hmox1, Gpx4*), pyroptosis (*Nlrp3, Gsdmd, Il18, Il1b*), apoptosis (*Bak, Bax, Casp3, Casp7*), and necroptosis markers (*Ripk3, Mlkl*), respectively, after treatment of cells for 24 h with 5 µM concentrations of DMSO as vehicle (Co), erlotinib (Erlo), crizotinib (crizo), and erastin (Eras), respectively; *Gapdh* was used as reference gene; n=3 independent experiments; means ± SEM; p values are given for comparisons to control (Co) using Browne-Forsythe and Welch ANOVA (Dunnett corrected).

(e) Representative live cell images representative of n=3 independent experiments after staining with BODIPY 581/591 C11. Color change from red to green indicates lipid peroxidation, a hallmark of ferroptosis; blue color denotes Hoechst; scale bar = 50 µm.

Fig. S19

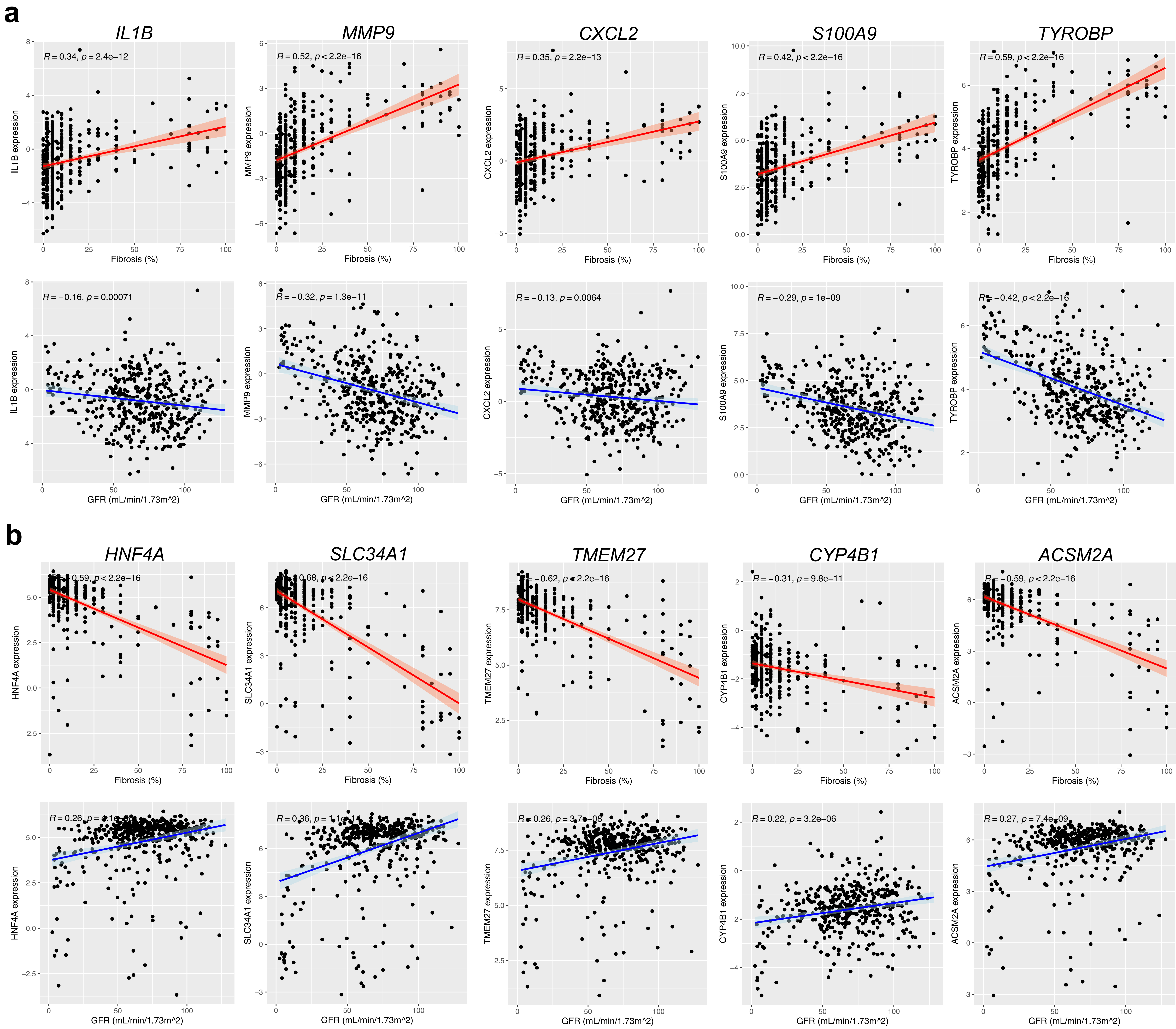
a
IL1B
R = 0.34, p = 2.4e−12
MMP9
R = 0.52, p < 2.2e−16
CXCL2
R = 0.35, p = 2.2e−13
S100A9
R = 0.42, p < 2.2e−16
TYROBP
R = 0.59, p < 2.2e−16
Fibrosis (%)
R = −0.16, p = 0.00071
R = −0.32, p = 1.3e−11
R = −0.13, p = 0.0064
R = −0.29, p = 1e−09
R = −0.42, p < 2.2e−16
GFR (mL/min/1.73m^2)
IL1B expression
MMP9 expression
CXCL2 expression
S100A9 expression
TYROBP expression
b
HNF4A
SLC34A1
TMEM27
CYP4B1
ACSM2A
R = −0.31, p = 9.8e−11
R = −0.59, p < 2.2e−16
R = 0.22, p = 3.2e−06
R = 0.27, p = 7.4e−09
HNF4A expression
SLC34A1 expression
TMEM27 expression
CYP4B1 expression
ACSM2A expression

Figure S19. Gene set enrichment analysis (GSEA) of maladaptive DEGs within drug response lists.

(a-b) Correlation of maladaptive repair (*IL1B, MMP9, CXCL2, S100A9, TYROBP*) **(a)** and successful repair gene (*HNF4A, SLC34A1, TMEM27, CYP4B1, ACSM2A*) **(b)** expression in microdissected human kidney tubules with fibrosis (top row) and eGFR (bottom row); error bands represent 95% confidence interval.

Fig. S20

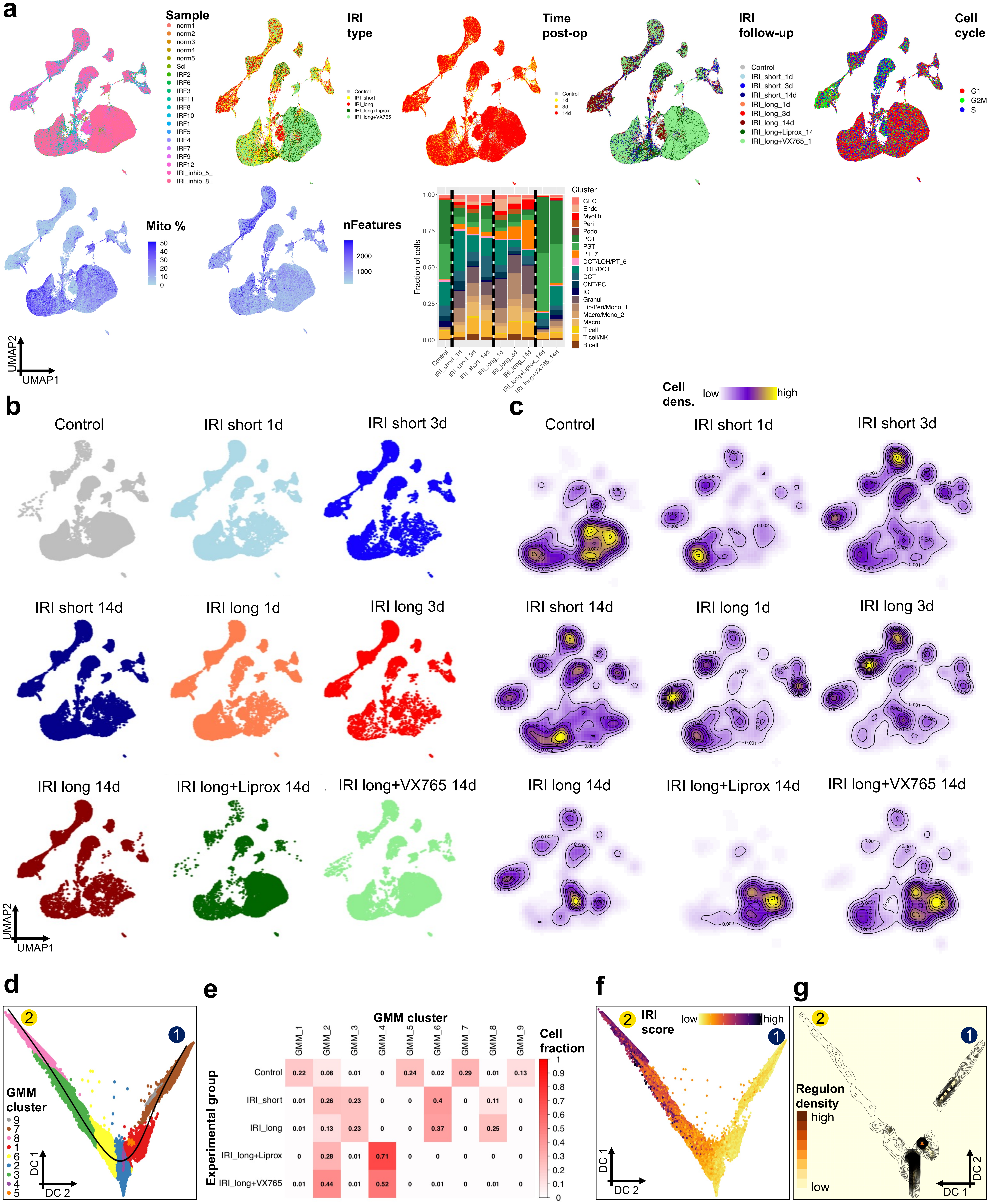
a
Sample
norm1
norm2
norm3
norm4
norm5
Scl
IRF2
IRF6
IRF3
IRF11
IRF8
IRF10
IRF1
IRF5
IRF4
IRF7
IRF9
IRF12
IRI_inhib_5_
IRI_inhib_8
IRI type
Control
IRI_short
IRI_long
IRI_long+Liprox
IRI_long+VX765
Time post-op
Control
1d
3d
14d
IRI follow-up
Control
IRI_short_1d
IRI_short_3d
IRI_short_14d
IRI_long_1d
IRI_long_3d
IRI_long_14d
IRI_long+Liprox_14
IRI_long+VX765_1
Cell cycle
G1
G2M
S
Mito %
nFeatures
UMAP2
UMAP1
Fraction of cells
Cluster
GEC
Endo
Myofib
Peri
Podo
PCT
PST
PT_7
DCT/LOH/PT_6
LOH/DCT
DCT
CNT/PC
IC
Granul
Fib/Peri/Mono_1
Macro/Mono_2
Macro
T cell
T cell/NK
B cell
b
Control
IRI short 1d
IRI short 3d
IRI short 14d
IRI long 1d
IRI long 3d
IRI long 14d
IRI long+Liprox 14d
IRI long+VX765 14d
c
Cell dens.
low
high
d
GMM cluster
DC 1
DC 2
e
GMM cluster
Experimental group
Cell fraction
Control: GMM_1 0.22, GMM_2 0.08, GMM_3 0.01, GMM_4 0, GMM_5 0.24, GMM_6 0.02, GMM_7 0.29, GMM_8 0.01, GMM_9 0.13
IRI_short: 0.01, 0.26, 0.23, 0, 0, 0.4, 0, 0.11, 0
IRI_long: 0.01, 0.13, 0.23, 0, 0, 0.37, 0, 0.25, 0
IRI_long+Liprox: 0, 0.28, 0.01, 0.71, 0, 0, 0, 0, 0
IRI_long+VX765: 0.01, 0.44, 0.01, 0.52, 0, 0.01, 0, 0.01, 0
f
IRI score
low
high
g
Regulon density
high
low
DC 1
DC 2

Figure S20. Pharmacological inhibition of pyroptosis and ferroptosis ameliorates maladaptive kidney response and fibrosis after severe IRI.

(a) UMAP projection of 133,433 kidney cells of Control, IRI short, IRI long and IRI long+VX765, and IRI long+Liproxstatin-treated samples. Coloring by sample, IRI type (Control, short IRI, long IRI, long IRI+VX765, long IRI+Liprox), time post-op (Control, 1d, 3d, 14d), IRI follow-up (Control, IRI short 1d, IRI short 3d, IRI short 14d, IRI long 1d, IRI long 3d, IRI long 14d, IRI long+VX765 14d, IRI long+Liprox 14d), cell cycle phase (G1, G2M, S), mitochondrial %, and number of features per cell. Last subpanel shows cell fraction across ischemia dose, time post-IRI, and treatment (Control, IRI short 1d, IRI short 3d, IRI short 14d, IRI long 1d, IRI long 3d, IRI long 14d, IRI long+VX765 14d, IRI long+Liprox 14d). Note reduction of PT_7_Maladaptive cells and general preservation of convoluted (PCT) and straight (PST) PT segments in inhibitor-treated samples.

(b) UMAP projections are split by treatment group for better viewability.

(c) Changes in cell type proportions of cell types between different treatment groups. Inhibitor-treated kidneys display cell densities similar to Control samples. Highest density in IRI long 14d kidneys corresponds to PT_7_Maladaptive.

(d) Diffusion map dimensional reduction representation of Slingshot-derived cell trajectories of 47,791 PT cells using a Gaussian mixture modeling (GMM) approach yielded 9 clusters in 2 trajectories. GMM clusters demonstrated major overlap with treatment groups (**Fig. 9h**). Inhibitor-treated samples overlapped with GMM clusters 2 and 4 at the junction right between lineages 1 and 2.

(e) Correlation matrix of cell fractions of GMM clusters across experimental groups, row sums equal 100%, identifying GMM clusters 2 and 4 as “drug response”-specific.

(f) Corresponding IRI scores projected onto diffusion map embedding space demonstrated a steady IRI score increase along trajectory 2, whereas IRI score was lowest in clusters from inhibitor-treated and Control samples.

(g) Corresponding SCENIC-derived regulon density is highest in clusters of inhibitor-treated samples at the junction of lineages 1 and 2. Corresponding tSNE visualization in **Fig. S21a**.

Fig. S21

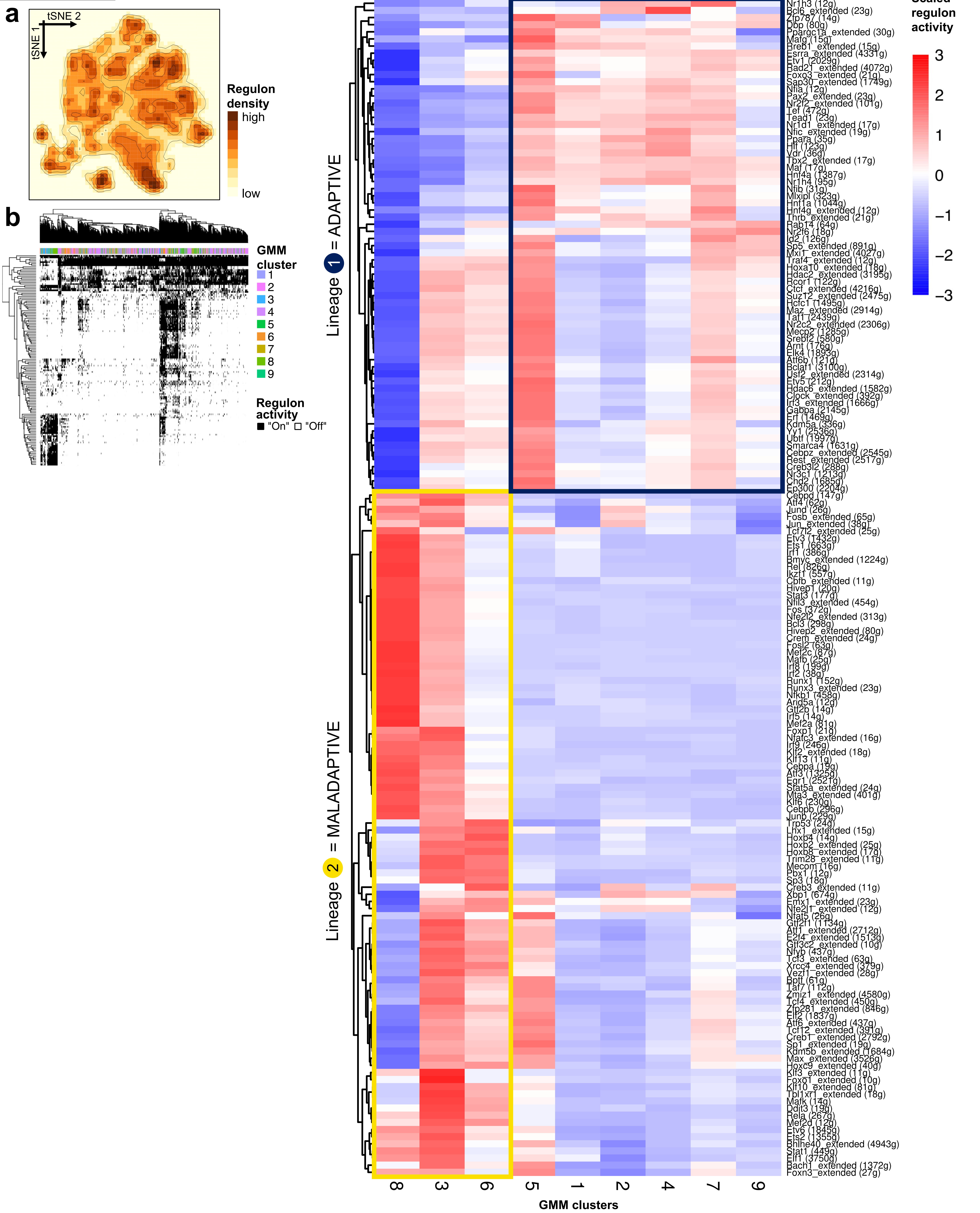
a
tSNE 2
tSNE 1
Regulon density
high
low
b
GMM cluster
1
2
3
4
5
6
7
8
9
Regulon activity
"On" "Off"
c
Scaled regulon activity
3
2
1
0
−1
−2
−3
Lineage 1 = ADAPTIVE
Lineage 2 = MALADAPTIVE
Nr1h3 (12g)
Bcl6_extended (23g)
Zfp787 (14g)
Dbp (80g)
Ppargc1a_extended (30g)
Mafg (15g)
Mafg (15g)
Nr1h3 (12g)
Bcl6_extended (23g)
Zfp787 (14g)
Dbp (80g)
Ppargc1a_extended (30g)
Mafg (15g)
Creb1_extended (15g)
Esrra_extended (4331g)
Etv1 (2029g)
Rad21_extended (4072g)
Foxo3_extended (21g)
Sap30_extended (1749g)
Nfia (12g)
Pax2_extended (23g)
Nr2f2_extended (101g)
Tef (472g)
Tead1 (23g)
Nr1d1_extended (17g)
Nfic_extended (19g)
Ppara (35g)
Hlf (123g)
Vdr (36g)
Tbx2_extended (17g)
Maf (17g)
Hnf4a (1387g)
Nr1h4 (95g)
Nfib (31g)
Mlxipl (323g)
Hnf1a (1044g)
Hnf4g_extended (12g)
Thrb_extended (21g)
Rab14 (64g)
Nr2f6 (18g)
Id2 (126g)
Sp5_extended (891g)
Mxi1_extended (4027g)
Traf4_extended (12g)
Hoxa10_extended (18g)
Hdac2_extended (3195g)
Rcor1 (122g)
Ctcf_extended (4216g)
Suz12_extended (2475g)
Hcfc1 (1495g)
Maz_extended (2914g)
Taf1 (2439g)
Nr2c2_extended (2306g)
Mecp2 (1285g)
Srebf2 (580g)
Arnt (176g)
Elk4 (1893g)
Atf6b (121g)
Bclaf1 (3100g)
Usf2_extended (2314g)
Etv5 (212g)
Hdac6_extended (1582g)
Clock_extended (392g)
Irf3_extended (1666g)
Gabpa (2145g)
Erf (1469g)
Kdm5a (336g)
Yy1 (2536g)
Ubtf (1997g)
Smarca4 (1631g)
Cebpz_extended (2545g)
Rest_extended (2517g)
Creb3l2 (288g)
Nr3c1 (1213g)
Chd2 (1685g)
Ep300 (2204g)
Cebpd (147g)
Atf4 (62g)
Jund (26g)
Fosb_extended (65g)
Jun_extended (38g)
Tcf7l2_extended (25g)
Etv3 (1432g)
Ets1 (663g)
Irf1 (386g)
Bmyc_extended (1224g)
Rel (826g)
Ikzf1 (557g)
Cbfb_extended (11g)
Hivep1 (20g)
Stat3 (177g)
Nfil3_extended (454g)
Fos (372g)
Nfe2l2_extended (313g)
Bcl3 (298g)
Hivep2_extended (80g)
Crem_extended (24g)
Fosl2 (63g)
Mef2c (87g)
Mafb (25g)
Irf8 (199g)
Irf2 (38g)
Runx1 (152g)
Runx3_extended (23g)
Nfkb1 (458g)
Arid5a (12g)
Gtf2b (14g)
Irf5 (14g)
Mef2a (81g)
Foxp1 (21g)
Nfatc3_extended (16g)
Irf9 (246g)
Klf2_extended (18g)
Klf13 (11g)
Cebpa (19g)
Atf3 (1325g)
Egr1 (2521g)
Stat5a_extended (24g)
Mta3_extended (401g)
Klf6 (230g)
Cebpb (296g)
Junb (229g)
Trp53 (24g)
Lhx1_extended (15g)
Hoxb4 (14g)
Hoxb2_extended (25g)
Hoxb8_extended (17g)
Trim28_extended (11g)
Mecom (16g)
Pbx1 (12g)
Sp3 (18g)
Creb3_extended (11g)
Xbp1 (674g)
Emx1_extended (23g)
Nfe2l1_extended (12g)
Nfat5 (26g)
Gtf2f1 (1134g)
Atf1_extended (2712g)
E2f4_extended (1513g)
Gtf3c2_extended (10g)
Nfyb (437g)
Tcf3_extended (63g)
Xrcc4_extended (379g)
Vezf1_extended (28g)
Bptf (61g)
Taf7 (112g)
Zmiz1_extended (4580g)
Tcf4_extended (450g)
Zfp281_extended (846g)
Elf2 (1837g)
Atf6_extended (437g)
Tcf12_extended (391g)
Creb1_extended (2792g)
Sp1_extended (19g)
Kdm5b_extended (1684g)
Max_extended (3526g)
Hoxc9_extended (40g)
Klf3_extended (11g)
Foxo1_extended (10g)
Klf10_extended (81g)
Tbl1xr1_extended (18g)
Mafk (14g)
Ddit3 (19g)
Rela (267g)
Mef2d (12g)
Etv6 (1845g)
Ets2 (1355g)
Bhlhe40_extended (4943g)
Stat1 (449g)
Elf1 (3750g)
Bach1_extended (1372g)
Foxn3_extended (27g)
8
3
6
5
1
2
4
7
9
GMM clusters

Figure S21. Gene regulatory network of pharmacological inhibition of pyroptosis and ferroptosis.

(a) tSNE representation of regulon density as a surrogate for stability of regulon states, as inferred by SCENIC algorithm, corresponding to diffusion map representation in **Fig. S20g**.

(b) Heatmap of cell type-specific binarized regulon activity, as inferred by SCENIC. Regulon activity was binarized to “on” (black) or “off” (white). Rows represent regulons, columns represent individual PT cells. Color legend at the top displays GMM clusters as derived from previous trajectory analysis in **Fig. S20d**.

(c) Heatmap of GMM cluster-specific scaled regulon activity. Rows represent regulons, columns represent GMM clusters as in **(b)**. GMM clusters 1, 2, 4, 5, 7, and 9 represented lineage 1, clusters 3, 6, and 8 lineage 2, respectively.

Full scan blots for Fig. S11d

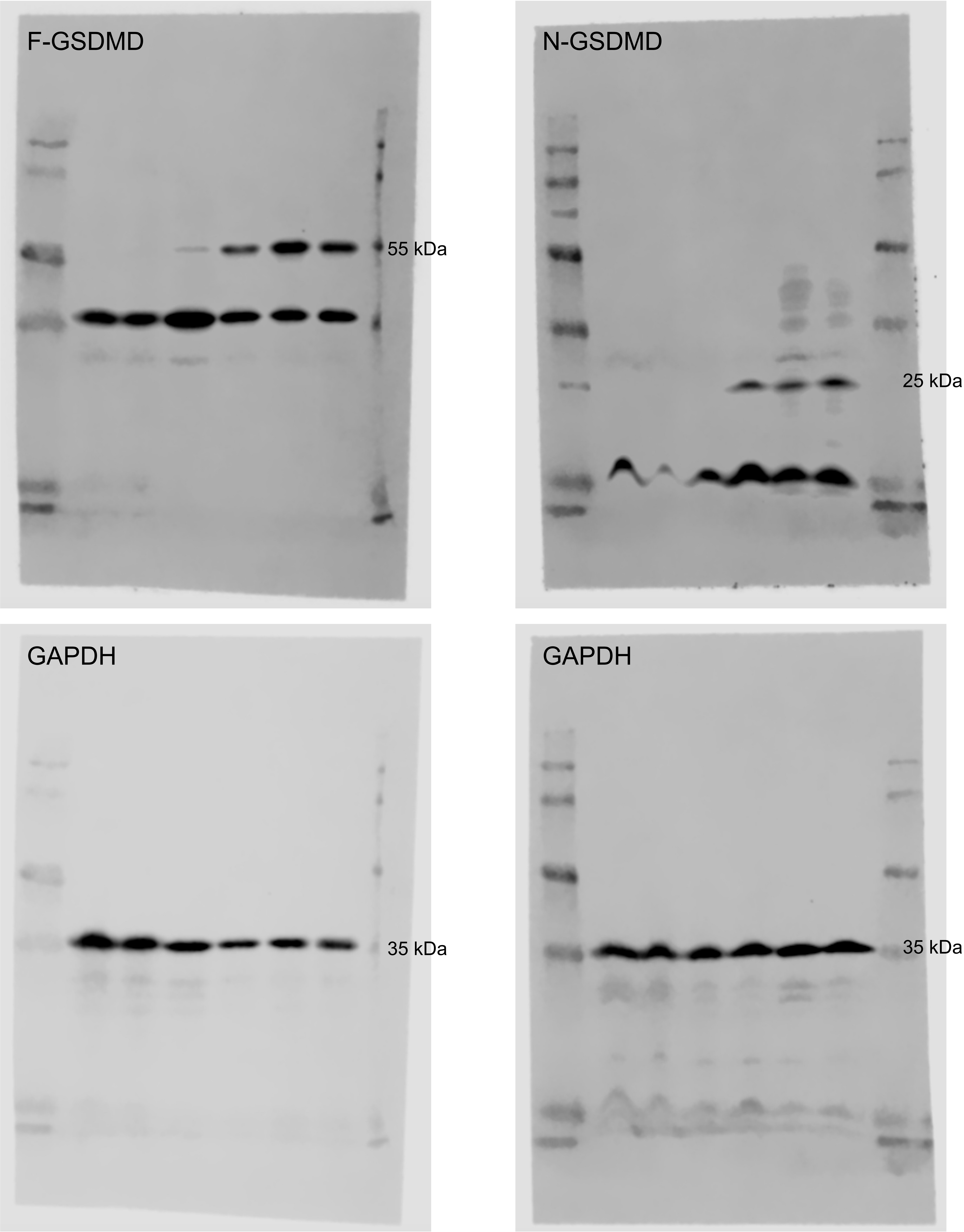

Supplementary Table 1. Flow cytometry antibodies.

Target	Dilution	Clone	Company	Fluorophore	Cat.#
CD3	1:200	145-2C11	BD bioscience	BUV395	100308
Ly6G	1:300	1A8	BD bioscience	BUV563	127602
CD11b	1:600	M1/70	BD bioscience	BUV661	612977
CD45	1:200	30-F11	BD bioscience	BUV805	103102
F4/80	1:200	BM8	Invitrogen	ef450	MF48000
CD19	1:300	6D5	Biolegend	BV605	115508
CD4	1:300	RM4-5	Biolegend	BV650	100506
NK1.1	1:200	PK136	Biolegend	BV711	108702
Ly6C	1:400	HK1.4	Biolegend	BV785	128016
CD11c	1:200	N418	eBioscience	FITC	117302
CD49b	1:100	DX5	eBioscience	PerCP-ef710	108908
SiglecF	1:200	E50-2440	BD bioscience	PE	562068
CD8a	1:300	53-6.7	BD bioscience	PE-cf594	562283
CD64	1:200	X54-5/7.1	Biolegend	PE-Cy7	139304
FcεR	1:200	MAR-1	Invitrogen	APC	17-5898-82
MHCII (IA/IE)	1:300	M5/114.15.2	Biolegend	AF700	107622
cKit (CD117)	1:200	ACK2	Invitrogen	APC-e780	47-1172-82

Supplementary Table 2. Materials table.

REAGENT or RESOURCE	SOURCE	IDENTIFIER
**Chemicals, Antibodies, Peptides, and Recombinant Proteins**		
Buprenorphine SR	midwest Veterinary Supply	191.26505.3
Liproxstatin	Cayman Chemical	17730
VX-765	InvivoGen	inh-vx765i-5
VetScan iSTAT CHEM8+	Abaxis	016.30270.2
DPBS	Corning	21-031-CV
RPMI 1640	Gibco	21875-034
Epithelial growth factor	Peprotech	AF-100-15
ITS	Gibco	51500--56
Penicillin-Streptomycin	Corning	30-002-CI
Dimethyl sulfoxide	Merck	D2650
Erlotinib	Cayman Chemical	10483
Crizotinib	Cayman Chemical	12087
Erastin	Cayman Chemical	17754
BODIPY™ 581/591 C11	Thermo Fisher	D3861
Hoechst 33342	Invitrogen	H1399
Tet System Approved FBS	Clontech	631106
Nonidet™ P40 Substitute	Sigma	74385
Magnesium Chloride Solution	Sigma	M1028
Ultrapure BSA (50 mg/ml)	Thermo Fisher	AM2616
Trizol	Invitrogen	15596018
RNAse inhibitor	Applied Biosystems	100021540
SYBR Green PCR Master Mix	Applied Biosystems	4367659
cDNA Reverse Transcription Kit	Applied Biosystems	4368813
RNAscope® 2.5 HD Duplex Detection Kit	ACD	322436
Mm-Gsdmd probe	ACD	537601
SDS lysis buffer	CST	7722
anti-GSDMD antibody	Santa Cruz	393656
anti-SLC34A1 antibody	Novus	NBP2-13328
anti-CD11B antibody	BD	555386
AF555-conj. donkey anti-rabbit	LifeSciences	A31572
AF488-conj. goat anti-mouse	LifeSciences	A11029
anti-GSDMD antibody	Abcam	ab209845
anti-N-GSDMD antibody	CST	36425S
HRP-conjugated secondary antibody	CST	7074
ECL SuperSignal West Femto	Thermo Fisher	34096
**Critical Commercial Assays**		
RNeasy mini kit	Qiagen	74106
Bioanalyzer High Sensitivity DNA kit	Agilent Technologies	5067-4626
Chromium Cell B Chip	10X Genomics	PN-120262
Chromium Controller	10X Genomics	PN-120223
Chromium i7 Multiplex Kit	10X Genomics	PN-120262
Chromium Single Cell 3’ Reagent Kits v3	10X Genomics	PN-1000092
Countess AutoCounter	Invitrogen	C10227
Illumina Library Quantification Kit	KAPA Biosystems	KK4824

Multi Tissue dissociation kit	Miltenyi	130-110-201
CytoTox 96 Non-Radioactive cytotoxicity assay	Promega	G1780
MultiTox-Fluor Multiplex Cytotoxicity assay	Promega	G9200
**Deposited Data**		
Mouse folic acid injury scRNA-seq data	(Dhillon et al.)	GSE156686 [https://www.ncbi.nlm.nih.gov/geo/query/acc.cgi?acc=GSE156686]
Mouse acute kidney injury snRNA-seq data	(Kirita et al.)	GSE139107 [https://www.ncbi.nlm.nih.gov/geo/query/acc.cgi?acc=GSE139107]
Mouse acute kidney injury bulk Affymetrix microarray data	(Liu et al.)	GSE52004 [https://www.ncbi.nlm.nih.gov/geo/query/acc.cgi]
Mouse kidnehy transplant scRNA-seq data	(Dangi et al.)	GSE157292 [https://www.ncbi.nlm.nih.gov/geo/query/acc.cgi]
Mouse acute spinal cord injury scRNA-seq data	(Hamel et al.)	GSE15638 [https://www.ncbi.nlm.nih.gov/geo/query/acc.cgi]
**Software and Algorithms**		
a4Base v1.34.1	open source	https://bioconductor.org/packages/release/bioc/html/a4Base.html
Biobase v2.40.0	open source	https://bioconductor.org/packages/release/bioc/html/Biobase.html
BisqueRNA v1.0.3	open source	https://cran.r-project.org/web/packages/BisqueRNA/index.html
Cell Ranger v3.1.0	10X Genomics	https://support.10xgenomics.com/single-cell-gene-expression/software/downloads/latest
CellPhoneDB v2.1.2	open source	https://www.cellphonedb.org
circlize v0.4.11	open source	https://github.com/jokergoo/circlize
Circos Table Viewer v0.63-9	open source	http://mkweb.bcgsc.ca/tableviewer/
clusterProfiler v3.10.1	open source	https://guangchuangyu.github.io/software/clusterProfiler/
DESeq2 v1.10.1	open source	https://bioconductor.org/packages/release/bioc/html/DESeq2.html
destiny v3.1.1	open source	https://github.com/theislab/destiny
DoubletFinder v2.0	open source	https://github.com/chris-mcginnis-ucsf/DoubletFinder
EnhancedVolcano v1.0.1	open source	https://bioconductor.org/packages/release/bioc/html/EnhancedVolcano.html
FlowJo v10.8	open source	https://www.flowjo.com
gam v1.20	open source	https://cran.r-project.org/web/packages/gam/index.html
GSEA v4.0.3	open source	http://www.gsea-msigdb.org/gsea/index.jsp
htmlwidgets v1.5.1	open source	https://github.com/ramnathv/htmlwidgets/releases
LIGER v0.5.0	open source	https://github.com/MacoskoLab/liger
mclust v5.4.6	open source	https://mclust-org.github.io/mclust/
monocle2 v2.14.0	open source	http://cole-trapnell-lab.github.io/monocle-release/
monocle3 v0.1.3	open source	https://cole-trapnell-lab.github.io/monocle3/
MRI fibrosis tool (unversioned)	open source	https://github.com/MontpellierRessourcesImagerie/imagej_macros_and_scripts/wiki/MRI_Fibrosis_Tool#myfootnote1
MuSiC v0.1.1	open source	https://github.com/xuranw/MuSiC
OneR v2.2	open source	https://cran.r-project.org/web/packages/OneR/index.html
pagoda2 v0.1.1	open source	https://github.com/kharchenkolab/pagoda2/releases
RSEM v1.3.0	open source	https://github.com/deweylab/RSEM
SCENIC v1.1.2.2	open source	https://aertslab.org/#scenic
Seurat v3.1.2	open source	https://satijalab.org/seurat/
SeuratWrappers v0.1.0	open source	https://github.com/satijalab/seurat-wrappers
Slingshot v1.6.1	open source	https://bioconductor.org/packages/release/bioc/html/slingshot.html
SoupX v1.4.5	open source	https://github.com/constantAmateur/SoupX
STAR v2.4.1d	open source	https://github.com/alexdobin/STAR
TradeSeq v1.2.01	open source	https://statomics.github.io/tradeSeq/index.html
VelocytoR v0.6	open source	https://github.com/velocyto-team/velocyto.R
WGCNA v1.70-3	open source	https://github.com/cran/WGCNA
xbioc v0.1.18	open source	https://rdrr.io/github/renozao/xbioc/
