# Supplementary material for: Single-cell analysis highlights differences in druggable pathways underlying adaptive or fibrotic kidney regeneration
Source: Nat Commun. 2022 Jul 11;13:4018. doi: 10.1038/s41467-022-31772-9 (PMC9276703; doi:10.1038/s41467-022-31772-9)
Supplement: Supplementary file 3 — Description of additional Supplementary File [file 41467_2022_31772_MOESM3_ESM.pdf]

Descriptions of Additional Supplementary Data files

Supplementary Dataset 1. Bulk RNAseq gene expression.

Supplementary Dataset 2. DEGs by cell clusters in all kidney cells.

Supplementary Dataset 3. Cell numbers and fractions.

Supplementary Dataset 4. DEGs by IRI degree for individual cell types.

Supplementary Dataset 5. DEGs by cell clusters in proximal tubule cells.

Supplementary Dataset 6. Regulons and predicted targets in proximal tubule cells.

Supplementary Dataset 7. DEGs by IRI degree and time post-IRI in proximal tubule cells.

Supplementary Dataset 8. KEGG pathway enrichment in proximal tubule cells.

Supplementary Dataset 9. WGCNA in proximal tubule metacells.

Supplementary Dataset 10. KEGG pathway enrichment in WGCNA modules.

Supplementary Dataset 11. qPCR primer pairs.

Supplementary Dataset 12. WGCNA in proximal tubule metacells

Supplementary Dataset 13. KEGG pathway enrichment in WGCNA modules

Supplementary Dataset 14. Primary mouse renal tubular epithelial cell culture

Supplementary Dataset 15. Long IRI+inhibitors model

Supplementary Dataset 16. qPCR primer pairs
