# Supplementary material for: Single-cell analysis highlights differences in druggable pathways underlying adaptive or fibrotic kidney regeneration
Source: Nat Commun. 2022 Jul 11;13:4018. doi: 10.1038/s41467-022-31772-9 (PMC9276703; doi:10.1038/s41467-022-31772-9)
Supplement: Supplementary file 20 — Reporting Summary [file 41467_2022_31772_MOESM20_ESM.pdf]

nature portfolio

Corresponding author(s): Katalin Susztak

Last updated by author(s): Jun 21, 2022

Reporting Summary

Nature Portfolio wishes to improve the reproducibility of the work that we publish. This form provides structure for consistency and transparency in reporting. For further information on Nature Portfolio policies, see our Editorial Policies and the Editorial Policy Checklist.

Statistics

For all statistical analyses, confirm that the following items are present in the figure legend, table legend, main text, or Methods section.

n/a	Confirmed	
☐	☒	The exact sample size (*n*) for each experimental group/condition, given as a discrete number and unit of measurement
☐	☒	A statement on whether measurements were taken from distinct samples or whether the same sample was measured repeatedly
☐	☒	The statistical test(s) used AND whether they are one- or two-sided<br>*Only common tests should be described solely by name; describe more complex techniques in the Methods section.*
☒	☐	A description of all covariates tested
☐	☒	A description of any assumptions or corrections, such as tests of normality and adjustment for multiple comparisons
☐	☒	A full description of the statistical parameters including central tendency (e.g. means) or other basic estimates (e.g. regression coefficient) AND variation (e.g. standard deviation) or associated estimates of uncertainty (e.g. confidence intervals)
☐	☒	For null hypothesis testing, the test statistic (e.g. *F*, *t*, *r*) with confidence intervals, effect sizes, degrees of freedom and *P* value noted<br>*Give P values as exact values whenever suitable.*
☒	☐	For Bayesian analysis, information on the choice of priors and Markov chain Monte Carlo settings
☒	☐	For hierarchical and complex designs, identification of the appropriate level for tests and full reporting of outcomes
☐	☒	Estimates of effect sizes (e.g. Cohen's *d*, Pearson's *r*), indicating how they were calculated

Our web collection on statistics for biologists contains articles on many of the points above.

Software and code

Policy information about availability of computer code

Data collection

Cell Ranger v. 3.1.0 – Barcode Identification, Alignment, Filter, Deduplication

Data analysis

a4Base v1.34.1
Biobase v2.40.0
BisqueRNA v1.0.3
Cell Ranger v3.1.0
CellPhoneDB v2.1.2
circlize v0.4.11
Circos Table Viewer v0.63-9
clusterProfiler v3.10.1
DESeq2 v1.10.1
destiny v3.1.1
DoubletFinder v2.0
EnhancedVolcano v1.0.1
FlowJo v10.8

gam v1.20
GSEA v4.0.3
htmlwidgets v1.5.1
LIGER v0.5.0
mclust v5.4.6
monocle2 v2.14.0

monocle3 v0.1.3
MRI fibrosis tool (unversioned)
MuSiC v0.1.1
OneR v2.2
pagoda2 v0.1.1
RSEM v1.3.0
SCENIC v1.1.2.2
Seurat v3.1.2
SeuratWrappers v0.1.0
Slingshot v1.6.1
SoupX v1.4.5
STAR v2.4.1d
TradeSeq v1.2.01
VelocytoR v0.6
WGCNA v1.70-3
xbioc v0.1.18

Code available at: https://github.com/ms-balzer/IRI_adaptive_maladaptive_kidney_regeneration

For manuscripts utilizing custom algorithms or software that are central to the research but not yet described in published literature, software must be made available to editors and reviewers. We strongly encourage code deposition in a community repository (e.g. GitHub). See the Nature Portfolio guidelines for submitting code & software for further information.

Data

Policy information about availability of data

All manuscripts must include a data availability statement. This statement should provide the following information, where applicable:

- Accession codes, unique identifiers, or web links for publicly available datasets
- A description of any restrictions on data availability
- For clinical datasets or third party data, please ensure that the statement adheres to our policy

Raw and metadata are available at GEO accession number GSE180420 [https://www.ncbi.nlm.nih.gov/geo/query/acc.cgi?&acc=GSE180420]. Processed data are available via an interactive website (www.susztaklab.com/ischemia_reperfusion_injury/scRNA/). Further information and requests for resources and reagents should be directed to and will be fulfilled by the lead contact: Katalin Susztak. Email: ksusztak@pennmedicine.upenn.edu.

Field-specific reporting

Please select the one below that is the best fit for your research. If you are not sure, read the appropriate sections before making your selection.

☒ Life sciences ☐ Behavioural & social sciences ☐ Ecological, evolutionary & environmental sciences

For a reference copy of the document with all sections, see nature.com/documents/nr-reporting-summary-flat.pdf

Life sciences study design

All studies must disclose on these points even when the disclosure is negative.

Sample size	We compared the number of cells profiled in other published datasets to choose an adequate sample size for this study. We chose to at least the same or more animals in our study conmpared to prior single-cell studies in the same organ with a similar disease model. No statistical methods were used to pre-determine sample size. Post-hoc analysis showed high reproducibility and agreement of cell type clusters.
Data exclusions	No data were excluded in this study.
Replication	In vivo studies employed 2-3 independent experiments, as stated in the respective sections. In vitro studies employed 2-5 independent experiments. In silico studies were computed with a set seed to facilitate reproducibility.
Randomization	Purchased wild type mice were randomly assigned to experimental treatment groups.
Blinding	Blinding was not relevant to this type of analysis, we collected samples that were available to us. Investigators were blinded to allocation during experiments and outcome assessments.

Reporting for specific materials, systems and methods

We require information from authors about some types of materials, experimental systems and methods used in many studies. Here, indicate whether each material, system or method listed is relevant to your study. If you are not sure if a list item applies to your research, read the appropriate section before selecting a response.

Materials & experimental systems

n/a	Involved in the study	
[ ]	[x]	Antibodies
[x]	[ ]	Eukaryotic cell lines
[x]	[ ]	Palaeontology and archaeology
[ ]	[x]	Animals and other organisms
[x]	[ ]	Human research participants
[x]	[ ]	Clinical data
[x]	[ ]	Dual use research of concern

Methods

n/a	Involved in the study	
[x]	[ ]	ChIP-seq
[ ]	[x]	Flow cytometry
[x]	[ ]	MRI-based neuroimaging

Antibodies

Antibodies used

Target Dilution Clone Company Fluorophore Cat.#
CD3 1:200 145-2C11 BD bioscience BUV395 100308
Ly6G 1:300 1A8 BD bioscience BUV563 127602
CD11b 1:600 M1/70 BD bioscience BUV661 612977
CD45 1:200 30-F11 BD bioscience BUV805 103102
F4/80 1:200 BM8 Invitrogen ef450 MF48000
CD19 1:300 6D5 Biolegend BV605 115508
CD4 1:300 RM4-5 Biolegend BV650 100506
NK1.1 1:200 PK136 Biolegend BV711 108702
Ly6C 1:400 HK1.4 Biolegend BV785 128016
CD11c 1:200 N418 eBioscience FITC 117302
CD49b 1:100 DX5 eBioscience PerCP-ef710 108908
SiglecF 1:200 E50-2440 BD bioscience PE 562068
CD8a 1:300 53-6.7 BD bioscience PE-cf594 562283
CD64 1:200 X54-5/7.1 Biolegend PE-Cy7 139304
FcεR 1:200 MAR-1 Invitrogen APC 17-5898-82
MHCII (IA/IE) 1:300 M5/114.15.2 Biolegend AF700 107622
cKit (CD117) 1:200 ACK2 Invitrogen APC-e780 47-1172-82

Target Dilution Company Cat.#
anti-GSDMD antibody 1:100 Santa Cruz 393656
anti-SLC34A1 antibody 1:100 Novus NBP2-13328
anti-CD11B antibody 1:50 BD 555386
AF555-conj. donkey anti-rabbit 1:1,000 LifeSciences A31572
AF488-conj. goat anti-mouse 1:1,000 LifeSciences A11029
anti-GSDMD antibody 1:1,000 Abcam ab209845
anti-N-GSDMD antibody 1:1,000 CST 36425S
HRP-conjugated anti-rabbit antibody 1:2,000 CST 7074

Validation

All commercially available antibodies listed above were validated by manufacturers as documented on the website of the respective companies.

Animals and other organisms

Policy information about studies involving animals; ARRIVE guidelines recommended for reporting animal research

Laboratory animals

Male C57Bl/6J mice were obtained from Jackson Laboratories (Bar Harbor, ME, stock no. 000664). 10-12 weeks old mice were used for experiments. Some animals were randomly assigned to receive liproxstatin (Cayman Chemical Cat#17730) or VX-765 (InvivoGen Cat#inh-vx765i-5). Mice were housed in the Institute pathogen free animal house (12 h dark/light cycle) and fed with standard mouse diet and water ad libitum.

Wild animals

This study did not involve wild animals.

Field-collected samples

This study did not involve field-collected samples.

Ethics oversight

The animal experiments were reviewed and approved by the Institutional Animal Care and Use Committee (IACUC) of the University of Pennsylvania in accordance with the guidelines of the National Institutes of Health.

Note that full information on the approval of the study protocol must also be provided in the manuscript.

Flow Cytometry

Plots

Confirm that:

- [x] The axis labels state the marker and fluorochrome used (e.g. CD4-FITC).
- [x] The axis scales are clearly visible. Include numbers along axes only for bottom left plot of group (a 'group' is an analysis of identical markers).
- [x] All plots are contour plots with outliers or pseudocolor plots.
- [x] A numerical value for number of cells or percentage (with statistics) is provided.

Methodology

Sample preparation	Mice were perfused with 10 mL PBS prior to organ procurement. Kidneys were diced and digested in a solution of 1 mg/mL collagenase A (Roche) and 100 mg/mL DNase (Roche) in complete RPMI for 60 min at 37 °C to obtain a single cell suspension. Cells were homogenized by passing through an 18G needle 5 times and filtered through a 100 µm mesh. Cells were then washed with FACS buffer (1x PBS, 2.5% FBS, 2 mM EDTA) before incubating with Fc block (99.5% FACS buffer, 0.5% normal rat serum, 1 µg/mL 2.4G2 IgG antibody) prior to staining. Cells were stained with the viability dye Ghost Dye Violet 510 (Tonbo biosciences, #12-0870) and antibodies were used for subsequent staining.
Instrument	FACSymphony A3 (BD Biosciences)
Software	FlowJo v10.8 (TreeStar)
Cell population abundance	We used established immune cell sorting procedures.
Gating strategy	All cells were sort-purified using a FACSAriaII (BD Bioscience) as follows:<br>B cells: CD19+, CD3-<br>CD4 T cells: CD19-, CD3+, CD4+<br>CD8 T cells: CD19-, CD3+, CD8a+<br>NK cells: CD19-, CD3-, NK1.1+<br>Macrophages: CD19-, CD3-, NK1.1-, F4/80+, CD64+<br>Neutrophils: CD19-, CD3-, NK1.1-, Ly6G+, Ly6C+<br>Monocytes: CD19-, CD3-, NK1.1-, Ly6G-, Ly6C+<br>Basophils: CD19-, CD3-, NK1.1-, Ly6G-, Ly6C-, CD45int, CD49b+, FcEr+<br>Eosinophils: CD19-, CD3-, NK1.1-, Ly6G-, Ly6C-, CD45+, CD49b-, SiglecF+<br>Mast cells: CD19-, CD3-, NK1.1-, Ly6G-, Ly6C-, CD45+, CD49b-, SiglecF-, cKit+<br>Dendritic cells: CD19-, CD3-, NK1.1-, Ly6G-, Ly6C-, CD45+, CD49b-, SiglecF-, CKit-, CD11c+, MHCII+<br>Purity for all cell populations was determined to be 98% or higher.

- [x] Tick this box to confirm that a figure exemplifying the gating strategy is provided in the Supplementary Information.
